# Characterization of global and regional left heart function using advanced echocardiography following intravenous administration of pimobendan in awake dogs

**DOI:** 10.3389/fvets.2026.1842861

**Published:** 2026-07-03

**Authors:** Pierre Foulex, Valérie Chetboul, Emilie Moisset, Fany Roncin, Alona Dougère, Guillaume Noël, Emilie Tréhiou, Céline Pouzot-Nevoret, Valentin Bondoux, Mathieu Magnin

**Affiliations:** 1 Clinique vétérinaire Boulogne Roland-Garros, Boulogne-Billancourt, France; 2 Sudvetia, Aix-en-Provence, France; 3 Veranex France, Paris, France; 4 École Nationale Vétérinaire d’Alfort, CHUV-AC, Maisons-Alfort, France; 5 Université Paris Est Créteil, INSERM, IMRB, Créteil, France; 6 Université de Lyon, Vetagro Sup, Unité de Physiologie, Pharmacodynamie et Thérapeutique, Marcy-l’Étoile, France; 7 Université de Lyon, APCSe Agressions Pulmonaires et Circulatoires dans le Sepsis (RSNR 201622884J), VetAgro Sup, Marcy-l’Étoile, France; 8 Université de Lyon, VetAgro Sup, Biovivo/Institut Claude Bourgelat, Marcy-l’Étoile, France; 9 Université de Lyon, VetAgro Sup, Service de cardiologie, Marcy-l’Étoile, France; 10 Intensive Care Unit (SIAMU), Université de Lyon, VetAgro Sup, APCSe, Marcy-l’Étoile, France; 11 Université de Lyon, VetAgro Sup, Unité de Pharmacie et de Toxicologie, Marcy-l’Étoile, France

**Keywords:** dog, myocardial strain, pimobendan, speckle-tracking echocardiography, stroke volume, tissue doppler imaging

## Abstract

**Background:**

Pimobendan is commonly used in veterinary cardiology; however, its impact on myocardial deformation in conscious dogs over time remains poorly characterized.

**Objectives:**

This exploratory and descriptive study aimed to characterize the effects of intravenous pimobendan on left heart function using advanced echocardiography and to identify the myocardial deformation component most closely associated with stroke volume increase.

**Methods:**

Five healthy, conscious, male Beagle dogs received a single bolus of pimobendan. Conventional echocardiography, tissue Doppler imaging, and 2D speckle-tracking echocardiography were performed at baseline and up to 24 h post-administration. Longitudinal, circumferential, and radial left ventricular strain were assessed, along with left atrial function, stroke volume, and cardiac output.

**Results:**

Pimobendan induced a significant increase in left ventricular systolic performance. Radial strain increased markedly from 30 min to 6 h, while circumferential strain increased transiently up to 4 h, primarily at the endocardial and mid-myocardial levels. Longitudinal strain increased from 30 min to 8 h, with a pronounced regional effect characterized by a greater enhancement of the apical segment, resulting in an accentuated base-to-apex strain gradient. Stroke volume increased significantly for up to 8 h and showed the strongest statistical association with longitudinal strain, particularly the apical component. Left atrial contractile function also improved transiently following treatment.

**Conclusion:**

Intravenous pimobendan produces a sustained improvement in left heart function in healthy, conscious, male Beagle dogs. The increase in stroke volume was most strongly associated with longitudinal myocardial deformation, especially at the apical level. Confirmation in females, other breeds, different age groups, and cardiac patients is warranted.

## Introduction

Advances in cardiac imaging have enabled increasingly detailed assessments of myocardial mechanics. Among these, two-dimensional speckle tracking echocardiography (2D STE) has emerged as a key tool for analyzing myocardial deformation (1–5). These techniques have demonstrated that left ventricular (LV) contraction is a complex, multidirectional process involving longitudinal shortening (resulting from reciprocal motion of the base toward the apex and the apex toward the base), radial thickening (inward toward the LV cavity), circumferential shortening (reduction in circular perimeter of the LV short axis), and opposite rotational motions of the base and apex resulting in LV twist (5–8). In clinical settings, these techniques allow for the identification of disease-specific patterns of myocardial dysfunction. For instance, in human patients with myxomatous mitral valve disease (MMVD), longitudinal function is preferentially impaired (9). In dogs with the dilated cardiomyopathy (DCM) phenotype, all components of LV myocardial deformation are reduced, but the radial component appears to be the most severely affected, with impairment increasing from the LV base to the apex, suggesting a regional pattern of systolic dysfunction (10–12).

Pimobendan is a widely used drug in veterinary cardiology (13–16). By enhancing contractility and reducing afterload, pimobendan improves cardiac output and tissue perfusion (17–21). In addition, it has been shown to reduce the severity of mitral regurgitation (15, 22). The cardiovascular effects of pimobendan have been extensively studied in anesthetized healthy dogs (17, 18, 20, 23–25). However, data in awake animals remain scarce. Intravenous administration has been shown to increase conventional echocardiographic indices of LV and left atrial (LA) contractility within 15 min and up to 2 h post-injection (17, 23, 24), but its sustained effects beyond 2 h have not been fully characterized. Moreover, to the best of the authors’ knowledge, only one study has applied STE to evaluate myocardial strain after pimobendan administration. The study reported that radial strain increased significantly 30–60 min after treatment, whereas longitudinal and circumferential strain remained unchanged (23). Thus, important questions remain regarding the evolution of pimobendan’s effects over time in conscious dogs, particularly with respect to the distinct components of myocardial deformation.

We therefore conducted an exploratory and descriptive study in healthy, conscious, male Beagle dogs receiving a single intravenous dose of pimobendan. Using echocardiography, including conventional echo-Doppler, tissue Doppler imaging (TDI), and STE, we characterized the effects of the drug on left heart function and systemic vascular resistance over a 24-h period. Given the exploratory nature of this study, multiple outcome measures were assessed to describe the time course of pimobendan’s effects on LV systolic deformation as comprehensively as possible. Among these, and given that pimobendan is primarily known as a positive inotropic agent, one of our specific aims was to identify which component of myocardial deformation showed the strongest statistical association with the hemodynamic response, defined as an increase in stroke volume (SV).

## Materials and methods

### Ethical statement

This study was approved by the institution’s Ethics Committee (APAFIS#48007-2024021917014478v5). All procedures complied with the European Directive 2010/63/EU. This article was prepared in accordance with the ARRIVE 2.0 guidelines.

### Animals

The sample size was determined to ensure that the study could detect a 30% increase in cardiac parameters following pimobendan administration, based on data published elsewhere (20). Cardiac output was used in the calculation, with a pre-treatment value set at 3 L/min (26) (±0.3). The sample size calculation accounted for paired data, with a significance level of 0.05 and a statistical power of 0.90. Based on this calculation, a minimum of four healthy beagles was required. To account for potential exclusions, five dogs were included. To be included in the study, the dogs had to be clinically healthy male Beagles. Dogs were excluded if any procedure in the experimental protocol could not be performed or if they exhibited signs of illness prior to anesthesia for arterial catheter placement. A health assessment, including a clinical examination, biochemical profile, and hematological analysis, was conducted 3 days before the experiments began.

### Experimental protocol

This study was designed as a descriptive, exploratory experimental investigation aimed at characterizing the effects of pimobendan on left heart function in conscious dogs and providing a macroscopic, mechanistic explanation of its positive inotropic effect. The complete experimental protocol is detailed in Supplementary Method A. Briefly, the dogs received an intravenous bolus of pimobendan (0.15 mg/kg). Invasive arterial blood pressure, including systolic (SAP), mean (MAP), and diastolic (DAP) pressures, as well as electrocardiographic parameters, were continuously monitored for 8 h following drug administration. Clinical and echocardiographic examinations were performed at baseline (T0) and at multiple time points after pimobendan injection (T30min, T2h, T4h, T6h, T8h, and T24h). Arterial blood samples were collected at predefined time points to determine the plasma concentrations of pimobendan and its active metabolite, O-desmethylpimobendan (ODMP), using liquid chromatography–tandem mass spectrometry. The dogs were housed individually during the monitoring period and returned to their group housing after catheter removal, with a final clinical, echocardiographic, and pharmacokinetic evaluation performed at T24h.

### Echocardiography

A detailed description of the echocardiographic acquisition protocol and measurement procedures is provided in Supplementary Method B. Briefly, echocardiographic examinations were performed on standing dogs using a single ultrasound unit (Vivid IQ, GE Healthcare) equipped with phased-array transducers, as previously validated (27). Conventional two-dimensional (2D) and M-mode measurements included aortic (Ao) and LA diameters, which were measured at end-diastole (28) and end-systole (29) to calculate the LA:Ao ratio. Left atrial internal diameters at end-diastole (LAmin) and end-systole (LAmax) were also obtained to calculate LA fractional shortening (LAFS) (30). Left ventricular internal diameters at end-diastole (LVIDd) and end-systole (LVIDs) were measured to determine LV fractional shortening (LVFS) (31). E-point-to-septal separation (EPSS) was also assessed. Left ventricular volumes at end-diastole (LVDVol) and end-systole (LVSVol) were obtained using Simpson’s method of discs to calculate LV ejection fraction (LVEF) (32). Left atrial volumes at end-systole (LASVol) and end-diastole (LADVol) were measured using the monoplane Simpson’s method to determine left atrial ejection fraction (LAEF) (33). LV internal areas at end-diastole (LVIAd) and end-systole (LVIAs) were obtained to calculate LV fractional area change (LVFAC) (32).

Conventional Doppler measurements included peak early (E) and late (A) mitral inflow velocities to determine the E:A ratio, along with isovolumetric relaxation time (IVRT). Cardiac output was estimated by combining aortic diameter (AoD) and aortic velocity time integral (VTIAo) measurements to derive SV and cardiac output (CO) (34).

Tissue Doppler imaging (TDI) was used to measure peak systolic (S′), early diastolic (E′), and late diastolic (A′) velocities at the lateral (E′l, A′l, S′l) and septal (E′s, A′s, S′s) mitral annuli (35).

Myocardial deformation was assessed using two-dimensional speckle-tracking echocardiography (STE) to measure peak systolic strain in the radial (rSt), longitudinal (lSt), and circumferential (cSt) directions (12). Global radial strain (rStglobal) was calculated from six myocardial segments, and radial synchrony was assessed as the delay between the earliest and latest peak rSt. Longitudinal strain was assessed using apical views to obtain global values (lStglobal4ch, lStglobal5ch, and lStglobal2ch) and their mean (lStglobalmean), in addition to regional values at the basal (lStbasal), mid-ventricular (lStmid), and apical (lStapical) levels. Circumferential strain was measured at the endocardial (cStendo), mid-myocardial (cStmid), and epicardial (cStepi) layers and averaged to obtain global circumferential strain (cStglobal) (36). To enhance clarity, the abbreviations of the STE parameters are summarized in Table 1.

**Table 1:** Abbreviations and definitions of the speckle tracking echocardiography parameters used in the study.

Strain variable	Significance
rSt	Systolic radial strain
lSt	Systolic longitudinal strain
cSt	Systolic circumferential strain
rStglobal	Mean of the peak rSt of the six left ventricular segments
cStglobal	Mean of peak cStendo, cStmid and cStepi
lStglobal4ch	Mean of the peak lSt of the six left ventricular segments from the left apical four-chamber view
lStglobal5ch	Mean of the peak lSt of the six left ventricular segments from the left apical five-chamber view
lStglobal2ch	Mean of the peak lSt of the six left ventricular segments from the left apical two-chamber view
lStglobalmean	Mean of lStglobal4ch, lStglobal5ch and lStglobal2ch
lStbasal*	Peak lSt averaged from the basal septal and basal lateral segments
lStmid*	Peak lSt averaged from the mid septal and mid lateral segments
lStapical*	Peak lSt averaged from the apical septal and apical lateral segments
cStendo	Peak cSt assessed at the endocardial level
cStmid	Peak cSt assessed at the mid-myocardial level
cStepi	Peak cSt assessed at the epicardial level
lStbasalmean	Mean of lStbasal from the left apical two-, four-, and five-chamber views
lStmidmean	Mean of lStmid from the left apical two-, four-, and five-chamber views
lStapicalmean	Mean of lStapical from the left apical two-, four-, and five-chamber views

*For each parameter, a suffix was added to indicate the echocardiographic view from which the measurement was obtained (2ch for the two-chamber view, 4ch for the four-chamber view, and 5ch for the five-chamber view).

### Statistical analysis

Statistical analyses were performed using the RStudio software with the ‘ggplot2’ (37) and ‘lme4’ (38) packages. The significance threshold was set at 5%. Longitudinal data analysis was conducted using linear mixed models. In each model, the variable of interest was designated as the dependent variable, time (considered a categorical variable) was included as the independent variable (fixed effect), and the dog’s name was used as a random effect. These models allowed comparisons between the values of the variables of interest at each time point and the baseline value at T0. The robustness of this modeling choice was assessed for strain parameters in a sensitivity analysis that used natural cubic splines to model time as a continuous variable, which additionally allowed for the estimation of the pharmacodynamic Tmax for each strain parameter (Supplementary Sensitivity Analysis). To investigate the association between STE parameters and SV, a linear mixed model was constructed, with SV as the dependent variable, STE parameters as independent variables (fixed effects), and the dog’s name as a random effect. A similar model was used to assess the association between plasma concentrations of pimobendan, ODMP, and SV. Initially, a simple model was built that included the plasma concentration of each substance as an independent variable. Subsequently, a model incorporating both pimobendan and ODMP plasma concentrations as independent variables was developed. Restricted maximum likelihood was used to estimate the variance and covariance parameters of the models. For each model, homoscedasticity and random distribution of residuals were examined by plotting residuals against fitted values. The model results are presented with the estimate (regression coefficient) and its 95% confidence interval. The results were considered statistically significant if zero was not included in the 95% confidence interval of the estimate. To compare the variations observed across the different components of longitudinal strain, a Kruskal-Wallis test was performed at each time point. When a significant difference was detected, *post hoc* pairwise comparisons were conducted using Dunn’s test with Holm’s correction for multiple testing.

## Results

The five dogs included in the study were male Beagles, aged 3 years at the time of the experiment, and their body weights ranged from 8 to 12 kg. No dogs were excluded.

### Pimobendan and ODMP concentrations

**Figure 1:**
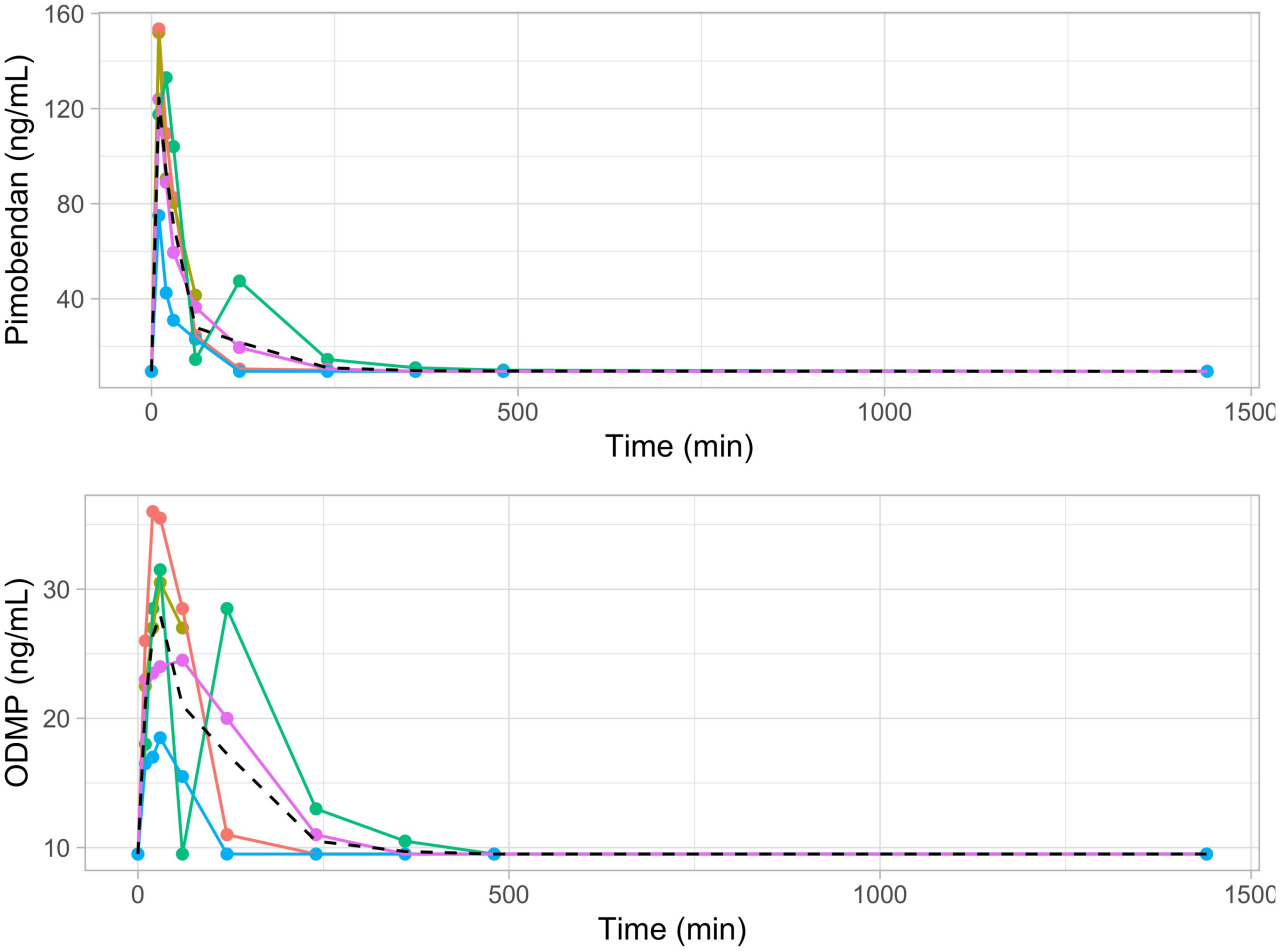
Evolution of plasma concentrations of pimobendan and O-desmethylpimobendan over time after intravenous pimobendan administration in five healthy, awake Beagle dogs. Pimobendan (0.15 mg/kg IV) was administered immediately after baseline (T0) measurements. Each color represents an individual dog. The dashed black line represents the mean. The lower limit of quantification was 10 ng/mL for both pimobendan and ODMP*. ODMP: O-desmethylpimobendan.*

A rapid increase in plasma concentrations of pimobendan and ODMP was observed, followed by a swift decline. ODMP appeared to persist longer in the bloodstream. Overall, the concentration–time profiles of both pimobendan and ODMP were relatively consistent across individuals, with the exception of a single, unexplained aberrant ODMP value of 10 ng/mL (the lower limit of quantification) at T30min (Figure 1, Supplementary Table A).

### Systolic function

#### Left ventricle

##### Conventional echocardiography

**Figure 2:**
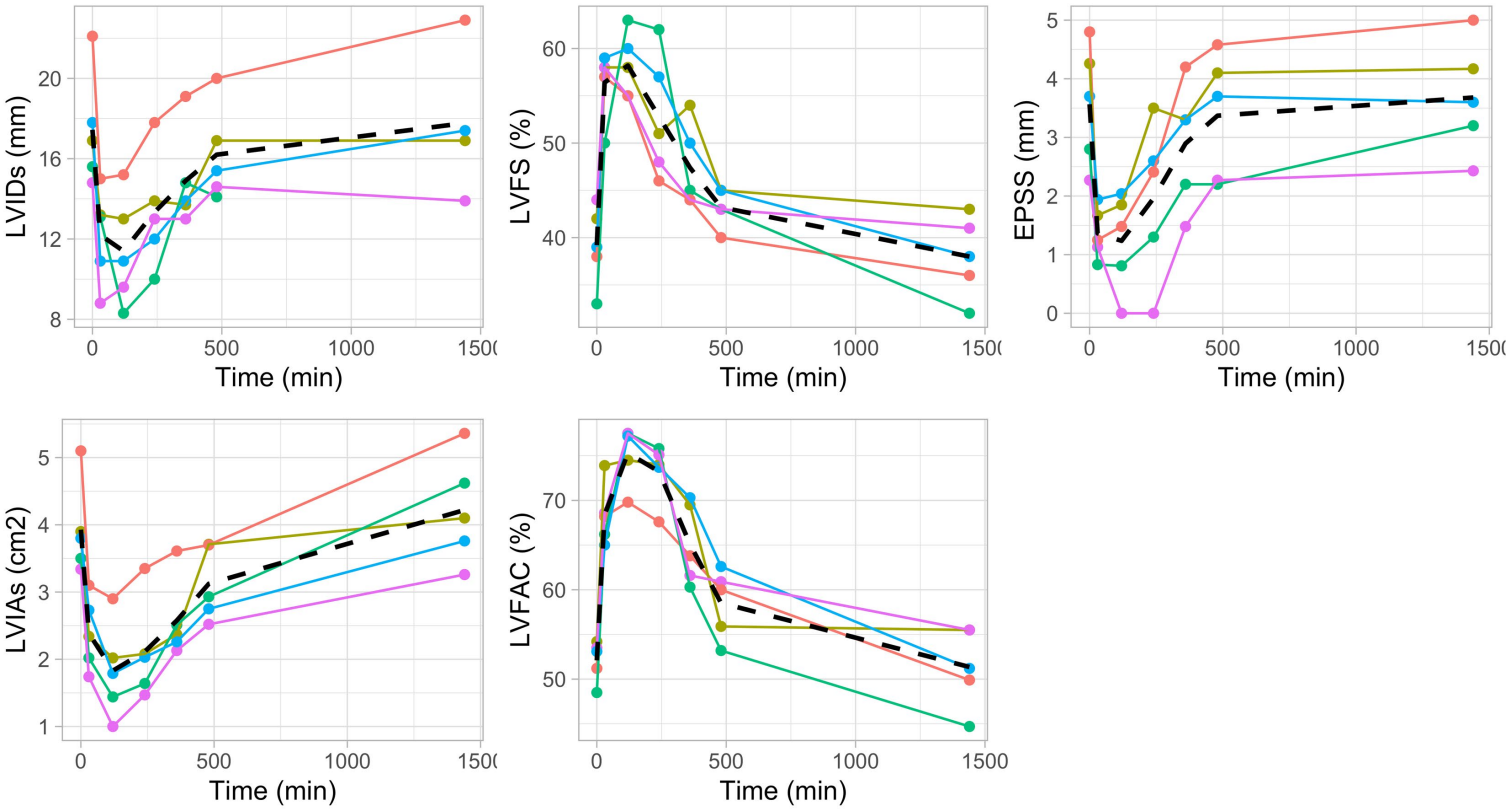
Evolution of left heart radial and circumferential parameters over time, as assessed by conventional echocardiography following intravenous pimobendan administration in five healthy, awake Beagle dogs. Pimobendan (0.15 mg/kg IV) was administered immediately after baseline (T0) measurements. Each color represents an individual dog. The dashed black line represents the mean. LVIDs, left ventricular internal diameter at end-systole; LVFS, left ventricular fractional shortening; EPSS, E-point to septal separation; LVIAs, end-systolic LV internal area; LVFAC, left ventricular fractional area change.

*Radial and circumferential parameters*: A significant decrease in both LVIDs and EPSS and a significant concomitant increase in LVFS were observed between T30min and T6h, with maximal effects observed between T30min and T2h. Similarly, LVIAs decreased significantly, and LVFAC increased significantly between T30min and T8h, with maximal effects between T30min and T2h (Table 2, Figure 2, Supplementary Figure A).

**Table 2:** Radial and circumferential left ventricular parameters assessed at different time points using conventional echocardiography after intravenous pimobendan administration.

Dependent variable	Independent variable	Estimate	95% confidence interval
LVIDd (mm)	T30min	−0.60	−2.36; 1.16
	T2h	−1.62	−3.38; 0.14
	T4h	−0.62	−2.38; 1.14
	T6h	−0.32	−2.08; 1.44
	T8h	−0.42	−2.18; 1.34
	T24h	0.34	−1.42; 2.10
LVIDs (mm)	T30min	**−5.24**	**−6.63; −3.85**
	T2h	**−6.04**	**−7.43; −4.65**
	T4h	**−4.10**	**−5.49; −2.71**
	T6h	**−2.54**	**−3.93; −1.15**
	T8h	−1.24	−2.63; 0.15
	T24h	−0.05	−1.54; 1.44
LVFS (%)	T30min	**17**	**13; 22**
	T2h	**19**	**14; 24**
	T4h	**13**	**9; 18**
	T6h	**8**	**4; 13**
	T8h	4	−1; 9
	T24h	−1	−6; 3
EPSS (mm)	T30min	**−2.20**	**−2.76; −1.65**
	T2h	**−2.33**	**−2.89; −1.77**
	T4h	**−1.60**	**−2.16; −1.05**
	T6h	**−0.67**	**−1.23; −0.11**
	T8h	−0.19	−0.75; 0.36
	T24h	0.11	−0.44; 0.67
LVIAd (cm^2^)	T30 min	**−0.60**	**−1.19; −0.01**
	T2h	**−0.97**	**−1.56; −0.38**
	T4h	−0.46	−1.05; 0.13
	T6h	**−0.76**	**−1.35; −0.17**
	T8h	**−0.66**	**−1.25; −0.07**
	T24h	0.45	−0.14; 1.04
LVIAs (cm^2^)	T30min	**−1.54**	**−1.87; −1.21**
	T2h	**−2.09**	**−2.43; −1.77**
	T4h	**−1.81**	**−2.14; −1.49**
	T6h	**−1.35**	**−1.68; −1.02**
	T8h	**−0.81**	**−1.13; −0.48**
	T24h	0.29	−0.04; 0.62
LVFAC (%)	T30min	**16**	**13; 20**
	T2h	**23**	**19; 27**
	T4h	**21**	**17; 25**
	T6h	**13**	**9; 17**
	T8h	**6**	**3; 10**
	T24h	−1	−5; 3

Echocardiographic measurements were obtained at baseline (T0, before drug administration) and at 30 min (T30min), 2 h (T2h), 4 h (T4h), 6 h (T6h), 8 h (T8h), and 24 h (T24h) after intravenous injection of pimobendan in the five apparently healthy Beagle dogs included in the study. The values at the different time points were compared to those at T0 using a linear mixed model, with each dog’s name included as a random effect. In the table, the estimate represents the mean difference between each time point and T0. The estimate is presented along with its 95% confidence interval. Significant differences are indicated in bold. EPSS, E-point to septal separation; FS, fractional shortening; LVFAC, left ventricular fractional area change; LVIAd, end-diastolic left ventricular internal area; LVIAs, end-systolic left ventricular internal area, LVIDd, left ventricular internal diameter at end-diastole; LVIDs, left ventricular internal diameter at end-systole.

**Figure 3:**
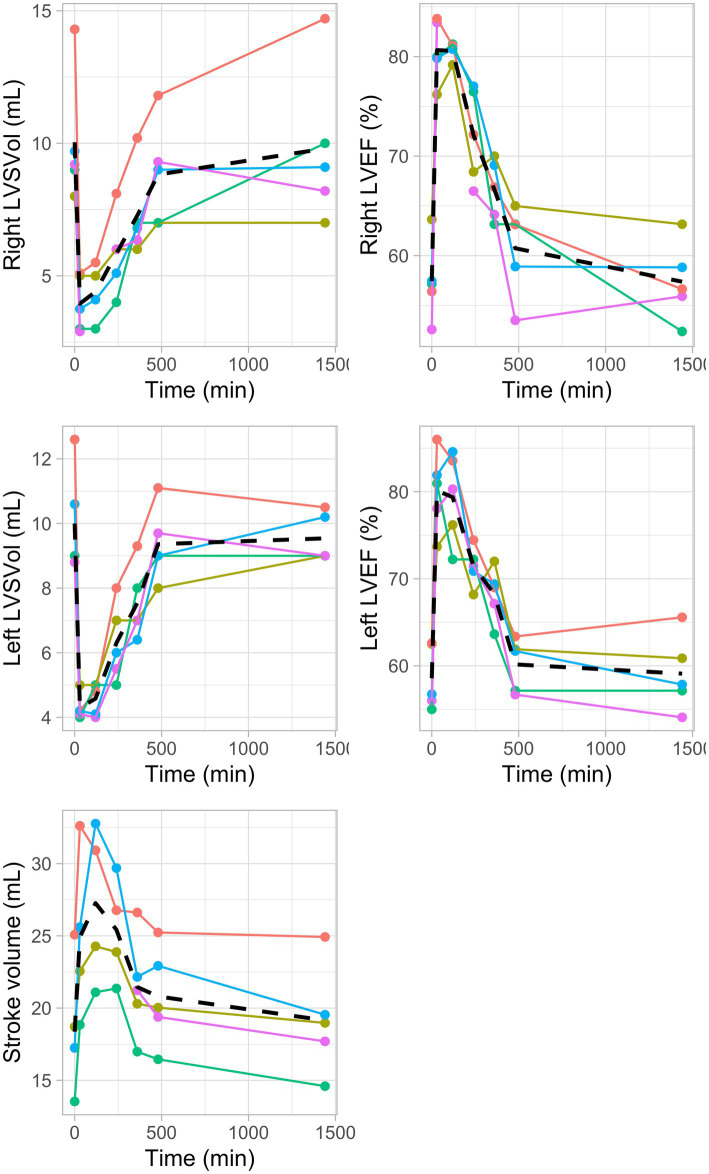
Evolution of left heart global parameters and stroke volume over time following intravenous pimobendan administration in five healthy, awake Beagle dogs. Pimobendan (0.15 mg/kg IV) was administered immediately after baseline (T0) measurements. Each color represents an individual dog. The dashed black line represents the mean. *Left LVSVol and Right LVSVol: left ventricular systolic volume assessed from the left apical and the right parasternal views, respectively; Left LVEF and Right LVEF: left ventricular ejection fraction assessed from the left apical and the right parasternal views, respectively.*

*Global parameters and stroke volume*: From both left apical and right parasternal views, a significant decrease in LVSVol and a significant concomitant increase in LVEF were observed between T30min and T6h, with the most pronounced changes occurring between T30min and T2h. Stroke volume significantly increased between T30min and T8h, with a peak at T2h. Changes in LVSVol and LVEF were very similar from both views over time (Table 3, Figure 3, Supplementary Figure B).

**Table 3:** Left ventricular volumes, ejection fraction, and stroke volume assessed by conventional echocardiography at different time points after intravenous pimobendan administration.

Dependent variable	Independent variable	Estimate	95% confidence interval
Right LVDVol (mL)	T30min	**−2.88**	**−4.68; −1.08**
	T2h	−1.89	−3.81, 0.04
	T4h	**−2.56**	**−4.36; −0.76**
	T6h	−1.70	−3.50; 0.10
	T8h	−1.02	−2.82; 0.78
	T24h	−0.68	−2.48; 1.12
Right LVSVol (mL)	T30min	**−6.09**	**−7.45; −4.73**
	T2h	**−5.78**	**−7.24; −4.33**
	T4h	**−4.20**	**−5.56; −2.84**
	T6h	**−2.77**	**−4.13; −1.41**
	T8h	−1.22	−2.58; 0.14
	T24h	−0.24	−1.60; 1.12
Right LVEF (%)	T30min	**23**	**19; 27**
	T2h	**23**	**19; 27**
	T4h	**15**	**11; 19**
	T6h	**9**	**5; 13**
	T8h	3	−1; 7
	T24h	0	−4; 4
Left LVDVol (mL)	T30min	**−2.06**	**−4.00; −0.12**
	T2h	−1.42	−3.36; 0.52
	T4h	**−2.20**	**−4.14; −0.26**
	T6h	−0.60	−2.54; 1.34
	T8h	−0.80	−2.74; 1.14
	T24h	−0.78	−2.72; 1.16
Left LVSVol (mL)	T30min	**−5.70**	**−6.64; −4.76**
	T2h	**−5.42**	**−6.36; −4.48**
	T4h	**−3.70**	**−4.64; −2.76**
	T6h	**−2.46**	**−3.40; −1.52**
	T8h	−0.64	−1.58; 0.30
	T24h	−0.46	−1.40; 0.48
Left LVEF (%)	T30min	**22**	**18; 25**
	T2h	**21**	**17; 24**
	T4h	**13**	**9; 16**
	T6h	**10**	**6; 13**
	T8h	2	−2; 5
	T24h	0	−3; 4
Stroke volume (mL)	T30min	**6**	**4; 18**
	T2h	**8**	**6; 10**
	T4h	**6**	**4; 8**
	T6h	**3**	**1; 5**
	T8h	**2**	**0.3; 4**
	T24h	0	−1; 2

Echocardiographic measurements were obtained at baseline (T0, before drug administration) and at 30 min (T30min), 2 h (T2h), 4 h (T4h), 6 h (T6h), 8 h (T8h), and 24 h (T24h) after intravenous injection of pimobendan in the five apparently healthy Beagle dogs included in the study. The values at the different time points were compared to those at T0 using a linear mixed model, with each dog’s name included as a random effect. In the table, the estimate represents the mean difference between each time point and T0. The estimate is presented along with its 95% confidence interval. Significant differences are indicated in bold. Left LVDVol, left ventricular end-diastolic volume measured from the left apical four-chamber view; Left LVEF, left ventricular ejection fraction assessed from the left apical four-chamber view; Left LVSVol, left ventricular end-systolic volume measured from the left apical four-chamber view; Right LVDVol, left ventricular end-diastolic volume measured from the right parasternal four-chamber view; Right LVEF, left ventricular ejection fraction assessed from the right parasternal four-chamber view; Right LVSVol, left ventricular end-systolic volume measured from the right parasternal four-chamber view.

##### TDI parameters

A significant increase in S’l was observed between T30min and T6h (Supplementary Table B and Supplementary Figure C).

##### STE parameters

No dogs were excluded at any time point due to insufficient image quality for STE analysis.

**Figure 4:**
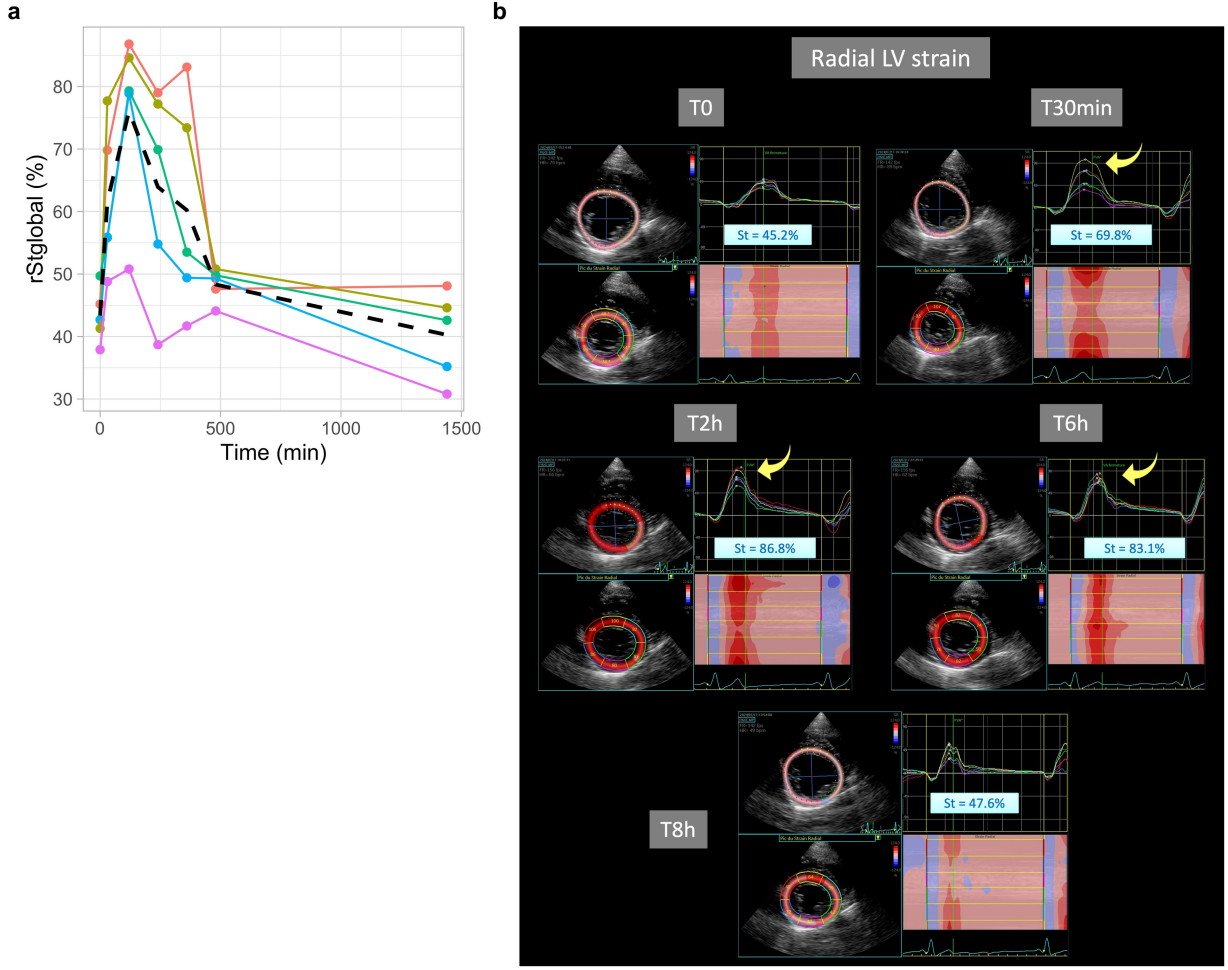
**(a)** Evolution of the global systolic radial strain (%) over time following intravenous pimobendan administration in five healthy, awake Beagle dogs. Pimobendan (0.15 mg/kg IV) was administered immediately after baseline (T0) measurements. Each color represents an individual dog. The dashed black line represents the mean. rStglobal is the mean of the systolic radial strains of the six left ventricular segments. **(b)** Representative evolution of left ventricular (LV) radial strain in one healthy dog included in the study following intravenous pimobendan administration. Radial strain was assessed using two-dimensional speckle-tracking echocardiography from the right parasternal transventricular short-axis view. Representative images obtained at baseline (T0) and at 30 min (T30min), 2 h (T2h), 6 h (T6h), and 8 h (T8h) after injection are shown. For each examination, the software automatically divided the LV myocardium into six equidistant segments distributed within the interventricular septum and the LV free wall. For each time point, LV short-axis segmentation (left panels), the corresponding color-coded strain M-mode display, and the radial strain-versus-time curves for the six myocardial segments (right panels) are presented. The lower left panels display the peak systolic strain values (%) obtained for each of the six myocardial segments. All six LV segments exhibit systolic myocardial thickening (positive strain), as illustrated on the two-dimensional short-axis images and on the color-coded M-mode display, where systolic deformation is represented in red. The value St (%) corresponds to the global peak systolic radial strain, calculated as the mean of the peak systolic strain values obtained in the six myocardial segments. Yellow arrows indicate time points at which peak systolic radial strain is increased compared with baseline. Following pimobendan administration, an increase in radial systolic myocardial deformation is observed, with maximal values between 2 and 6 h and a return toward baseline by 8 h.

*Radial systolic strain*: A significant increase in rStglobal was observed between T30min and T6h, with a peak at T2h. At T8h and T24h, the rSt parameters were close to the baseline values (Table 4 and Figure 4a). The increase in rStglobal was the result of the enhancement of all six segments, albeit to a slightly variable degree (Supplementary Table C and Supplementary Figure D). Figure 4b shows rSt variations in one dog from the study, from 45.2% at baseline before pimobendan injection (T0) to 86.8% at T2h, before decreasing to 47.6% at T8h, close to the baseline value.

**Table 4:** Global peak left ventricular systolic radial strain assessed by speckle tracking imaging from the right parasternal transventricular short-axis view after intravenous pimobendan administration.

Dependent variable	Independent Variable	Estimate	95% confidence interval
rStglobal (%)	T30min	**18**	**9; 27**
	T2h	**33**	**24; 42**
	T4h	**21**	**12; 29**
	T6h	**17**	**8; 26**
	T8h	5	−4; 14
	T24h	−3	−12; 6

Speckle tracking imaging measurements were obtained at baseline (T0, before drug administration) and at 30 min (T30min), 2 h (T2h), 4 h (T4h), 6 h (T6h), 8 h (T8h), and 24 h (T24h) after intravenous injection of pimobendan in the five apparently healthy Beagle dogs included in the study. The absolute values at the different time points were compared to those at T0 using a linear mixed model, with each dog’s name included as a random effect. In the table, the estimate represents the mean difference between each time point and T0. The estimate is presented along with its 95% confidence interval. Significant differences are indicated in bold. All strain values are expressed as absolute values; therefore, the estimates correspond to changes in the absolute magnitude of the strain parameters. rStglobal, global peak left ventricular systolic radial strain, defined as the mean peak systolic radial strain of the left ventricle, as assessed from six left ventricular myocardial segments on the right parasternal transventricular short-axis view.

**Figure 5:**
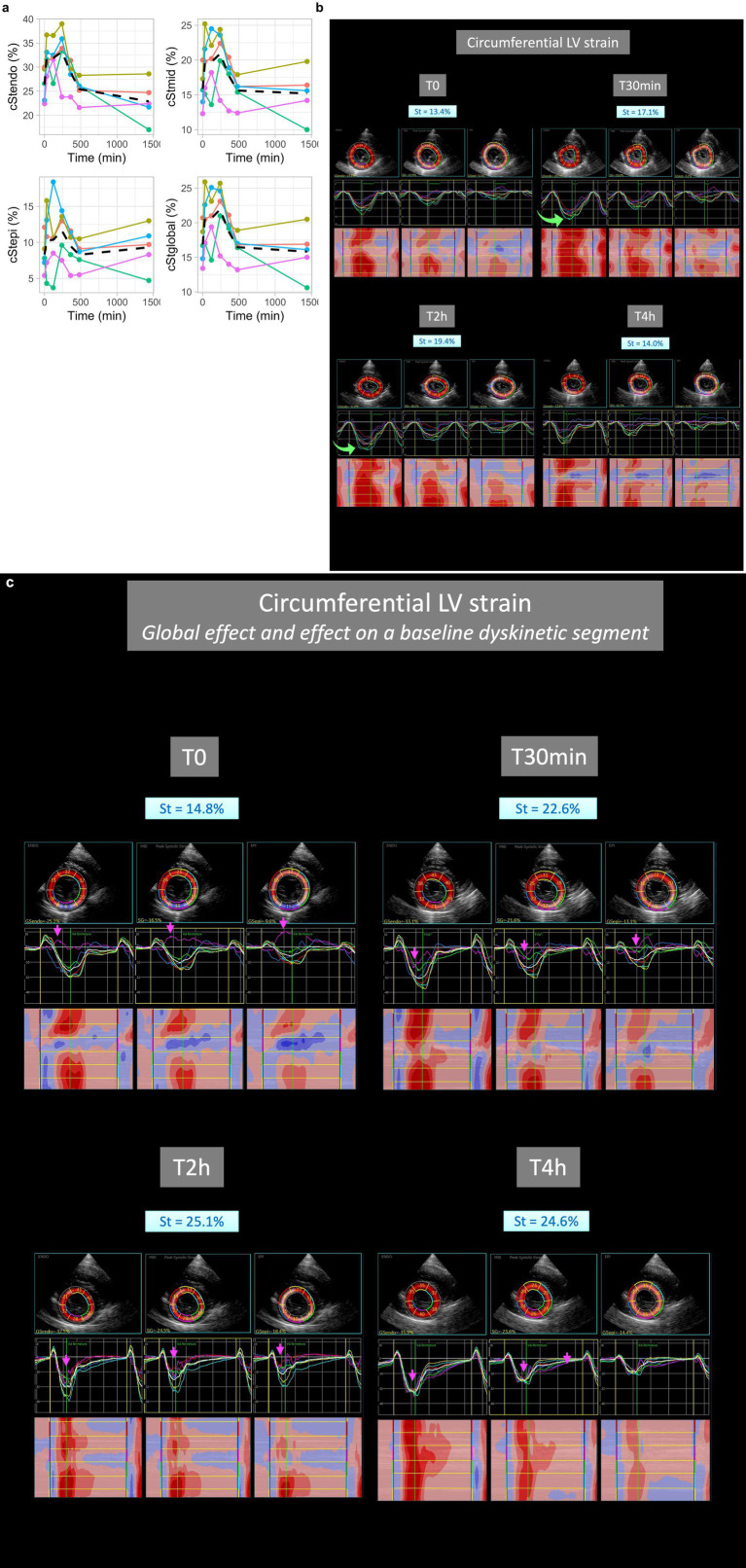
**(a)** Evolution of circumferential systolic strain parameters over time following intravenous pimobendan administration in five healthy, awake Beagle dogs. Pimobendan (0.15 mg/kg IV) was administered immediately after baseline (T0) measurements. Each color represents an individual dog. The dashed black line represents the mean. Absolute strain values were used. *cStendo, peak circumferential systolic strain evaluated at the endocardial level; cStmid, peak systolic circumferential strain evaluated at the midmyocardial level; cStepi, peak circumferential systolic strain evaluated at the epicardial level; cStglobal, the mean of the peak systolic circumferential strains evaluated at the endo-, mid-myocardial, and epicardial levels.* **(b)** Representative evolution of left ventricular (LV) circumferential strain in one healthy dog included in the study following intravenous pimobendan administration. Circumferential strain was assessed using the same two-dimensional speckle-tracking echocardiography technique from the same right parasternal short-axis view as described in Figure 4b. Representative images obtained at baseline (T0) and at 30 min (T30min), 2 h (T2h), and 4 h (T4h) after injection are shown. For each examination, the software automatically divided the LV myocardium into six equidistant segments. Peak systolic circumferential strain was independently evaluated at the endocardial (left panels), mid-myocardial (middle panels), and epicardial (right panels) segments. Circumferential strain reflects circumferential myocardial shortening (reduction of the LV circular perimeter in the short axis) and is therefore expressed as a negative value during systole. The upper panels display the peak systolic strain values obtained for each of the six myocardial segments, expressed as absolute values (%). The value St (%) corresponds to the mean absolute peak systolic circumferential strain calculated from the six myocardial segments within each myocardial layer. Green arrows indicate the time points corresponding to peak systolic circumferential strain in the endocardial layer, which shows the highest magnitude of deformation among the three myocardial layers. Following pimobendan administration, an increase in circumferential systolic myocardial deformation is observed, with maximal values around 2 h after injection. **(c)** Representative evolution of left ventricular (LV) circumferential strain in another healthy dog included in the study following intravenous pimobendan administration. Circumferential strain was assessed from the same right parasternal transventricular short-axis view using the same two-dimensional speckle-tracking echocardiography technique as described in Figure 4b. Representative images obtained at baseline (T0) and at 30 min (T30min), 2 h (T2h), and 4 h (T4h) after injection are shown. At baseline (T0), one myocardial segment located in the LV posterior wall shows dyskinetic behavior, characterized by circumferential elongation during systole (pink arrow), whereas the other myocardial segments exhibit normal circumferential shortening. Following pimobendan administration (T30min, T2h, and T4h), this segment becomes resynchronized with the other five myocardial segments and exhibits systolic circumferential shortening, contributing to an increase in the absolute value of global circumferential strain (St) from 14.8% at baseline to 25.1% at T2h (pink arrows).

*Circumferential systolic strain*: A significant increase in the absolute value of cStglobal was observed between 30 min and 4 h after pimobendan administration (Table 5 and Figure 5a). This increase was primarily driven by the elevation of cStendo and cStmid. Figure 5b highlights substantial interindividual variability. Figure 5b shows the variations in cSt in one dog in the study, with the absolute value of cStglobal increasing from 13.4% at baseline to reach 19.4% at T2h, before decreasing to a value close to the baseline at T8h (14.0%). Figure 5c shows the circumferential strain in another dog from the study, showing a dyskinetic myocardial segment (LV posterior segment) at baseline, before the pimobendan injection. This segment showed circumferential elongation during systole at T0, whereas all the others showed circumferential shortening. At T30min, T2h, and T4h after the pimobendan injection, this segment resynchronized with the other five segments and showed circumferential shortening, contributing to the increase in the absolute value of cStglobal from 14.8% at baseline to 25.1% at T2h.

**Table 5:** Global peak left ventricular systolic circumferential strain derived from endocardial, midmyocardial and epicardial circumferential strains assessed by speckle tracking imaging from the right parasternal transventricular short-axis view after intravenous pimobendan administration.

Dependent variable	Independent variable	Estimate	95% confidence interval
cStendo (%)	T30min	**6**	**3; 9**
	T2h	**6**	**2; 9**
	T4h	**7**	**4; 10**
	T6h	3	−0.5; 6
	T8h	−1	−4; 2
	T24h	**−3**	**−7; −0.4**
cStmid (%)	T30min	**4**	**1; 6**
	T2h	**4**	**1; 6**
	T4h	**5**	**2; 8**
	T6h	2	−1; 5
	T8h	0	−3; 2
	T24h	−1	−3; 2
cStepi (%)	T30min	2	−1; 5
	T2h	2	−1; 5
	T4h	**3**	**1; 6**
	T6h	1	−1; 4
	T8h	0	−3; 3
	T24h	1	−2; 4
cStglobal (%)	T30min	**4**	**1; 6**
	T2h	**4**	**1; 6**
	T4h	**5**	**3; 8**
	T6h	2	−1; 4
	T8h	0	−3; 2
	T24h	−1	−4; 2

Speckle tracking imaging measurements were obtained at baseline (T0, before drug administration) and at 30 min (T30min), 2 h (T2h), 4 h (T4h), 6 h (T6h), 8 h (T8h), and 24 h (T24h) after intravenous injection of pimobendan in the five apparently healthy Beagle dogs included in the study. The absolute values of circumferential strain at the different time points were compared to those at T0 using a linear mixed model, with each dog’s name included as a random effect. In the table, the estimate represents the mean difference between each time point and T0. The estimate is presented along with its 95% confidence interval. Significant differences are indicated in bold. All strain values are expressed as absolute values; therefore, the estimates correspond to changes in the absolute magnitude of strain parameters. cStendo, peak systolic circumferential strain at the endocardial level; cStepi, peak systolic circumferential strain at the epicardial level, cStmid, peak systolic circumferential strain at the midmyocardial level, cStglobal, the mean of peak cStendo, cStmid, and cStepi.

**Figure 6:**
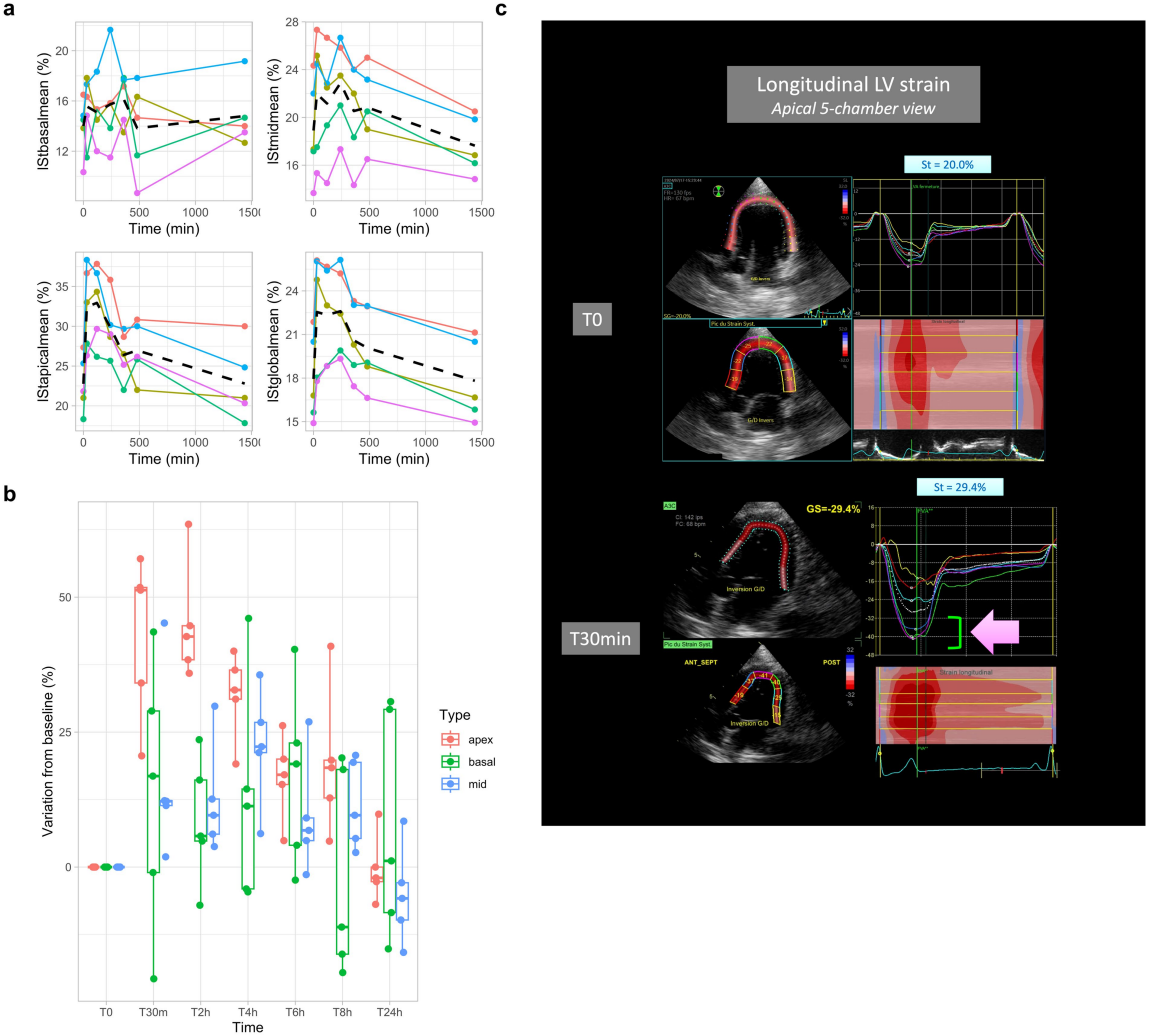
**(a)** Evolution of longitudinal systolic strain parameters over time following intravenous pimobendan administration in five healthy, awake Beagle dogs. Pimobendan (0.15 mg/kg IV) was administered immediately after baseline (T0) measurements. Each color represents an individual dog. The dashed black line represents the mean. Absolute strain values were used. lStbasalmean, mean of peak systolic longitudinal strain of basal left ventricular segments from the left apical four-, five- and two-chamber views; lStmidmean, mean of peak systolic longitudinal strain of mid left ventricular segments from the left apical four-, five- and two-chamber views; lStapicalmean, mean of peak systolic longitudinal strain of apical left ventricular segments from the left apical four-, five- and two-chamber views; lStglobalmean, mean of lStglobal2ch, lStglobal4ch and lStglobal5ch. **(b)** Variation from baseline of segmental longitudinal systolic strain (basal, mid, and apical) at all time points following intravenous pimobendan administration in five healthy, awake Beagle dogs. Pimobendan (0.15 mg/kg IV) was administered immediately after baseline (T0) measurements. Absolute strain values were used. Absolute strain values were used. Apex corresponds to lStapicalmean, basal corresponds to lStbasalmean, mid corresponds to lStmidmean. **(c)** Representative evolution of left ventricular (LV) longitudinal strain in a healthy dog included in the study following intravenous pimobendan administration. Longitudinal strain was assessed using two-dimensional speckle-tracking echocardiography from the left parasternal apical five-chamber view. Representative images obtained at baseline (T0) and at 30 min (T30min) after injection are shown. For each examination, the software automatically divided the LV myocardium into six longitudinal segments extending from the base to the apex. Color-coded myocardial segmentation is displayed on the two-dimensional images (left panels). The lower left panels display the peak systolic strain values obtained for each of the six myocardial segments, expressed as absolute values (%). The corresponding longitudinal strain-versus-time curves for the six myocardial segments are shown in the upper right panels, and the color-coded strain M-mode display is presented in the lower right panels. Longitudinal strain reflects systolic myocardial shortening along the long axis of the LV and is therefore normally expressed as negative values during systole. Following pimobendan administration, an early increase in the absolute value of global longitudinal strain (St) is observed, from 20.0% at baseline (T0) to 29.4% at T30min. This increase is heterogeneous across myocardial segments and is particularly marked in the LV apical segments large pink arrow, which exhibit greater systolic deformation than the basal segments.

*Longitudinal systolic strain*: A high degree of inter-individual variability and heterogeneity was observed among the different components of global longitudinal strain across all echocardiographic views (i.e., the left apical four-, five-, and two-chamber views). Details of the evolution of longitudinal strain parameters from each view are presented in Supplementary Tables D–F and Supplemetnary Figures E–G. A significant increase in the absolute value of lStglobalmean was observed between T30min and T8h. Similar patterns were found for lStmidmean and lStapicalmean, which increased significantly between T30min and T4h and between T30min and T8h, respectively, whereas lStbasalmean did not change significantly (Table 6 and Figure 6a). We observed a longitudinal strain gradient, with higher absolute values in lStapicalmean and lower values in lStbasalmean. This gradient was present at all study time points, but it appeared to become more pronounced following pimobendan administration (Figure 6b and Supplementary Table G and Supplementary Figures H,I). Statistical analysis confirmed significant differences in absolute longitudinal strain values at T30min and T2h between the three pairs of myocardial segments (Table 7). Moreover, at T2h, the increase in absolute lStapicalmean values was significantly higher than the increase in absolute lStmidmean values (*p* = 0.027) and absolute lStbasalmean values (*p* = 0.014). Figure 6c shows the variation in the absolute lStglobal5ch values in one dog from the study, increasing from 20.0% at baseline before pimobendan injection (T0) to 29.4% at T30min, with a maximal increase occurring in the LV apical segments.

**Table 6:** Mean left ventricular longitudinal systolic strain assessed by speckle tracking imaging from the apical two-, four-, and five-chamber views, according to left ventricular level after intravenous pimobendan administration.

Dependent variable	Independent variable	Estimate	95% confidence interval
lStbasalmean (%)	T30min	2	−1; 4
	T2h	1	−1; 3
	T4h	2	−0.5; 4
	T6h	2	−0.1; 4
	T8h	0	−2; 2
	T24h	1	−1; 3
lStmidmean (%)	T30min	**3**	**1; 5**
	T2h	**2**	**1; 4**
	T4h	**4**	**2; 6**
	T6h	2	−0.1; 3
	T8h	**2**	**0.2; 4**
	T24h	−1	−3; 0.4
lStapicalmean (%)	T30min	**10**	**7; 12**
	T2h	**10**	**8; 13**
	T4h	**7**	**5; 10**
	T6h	**4**	**1; 6**
	T8h	**4**	**2; 7**
	T24h	0	−2; 2
lStglobalmean (%)	T30min	**5**	**3; 6**
	T2h	**4**	**3; 6**
	T4h	**5**	**4; 6**
	T6h	**3**	**2; 4**
	T8h	**2**	**1; 3**
	T24h	0	−1; 1

Speckle tracking imaging measurements were obtained at baseline (T0, before drug administration) and at 30 min (T30min), 2 h (T2h), 4 h (T4h), 6 h (T6h), 8 h (T8h), and 24 h (T24h) after intravenous injection of pimobendan in the five apparently healthy Beagle dogs included in the study. The absolute values of longitudinal systolic strain at the different time points were compared to those at T0 using a linear mixed model, with each dog’s name included as a random effect. In the table, the estimate represents the mean difference between each time point and T0. The estimate is presented along with its 95% confidence interval. Significant differences are indicated in bold. All strain values are expressed as absolute values; therefore, the estimates correspond to changes in the absolute magnitude of the strain parameters. lStapicalmean, mean of peak systolic longitudinal strain of the apical septal and apical lateral segments averaged from three left apical views; lStbasalmean, mean of peak systolic longitudinal strain of the basal septal and basal lateral segments averaged from three left apical views; lStmidmean, mean of peak systolic longitudinal strain of the mid septal and mid lateral segments averaged from three left apical views, lStglobalmean, mean of peak systolic longitudinal strain assessed in 18 left ventricular segments, i.e., 6 from the left apical four-chamber view, 6 from the left apical five-chamber view, and 6 from the left apical two-chamber view.

**Table 7:** Comparison between the different components of systolic longitudinal strain according to the left ventricular level, as assessed by speckle tracking imaging after administration of intravenous pimobendan.

Traduce by time	Type	Variation from baseline (%)	Kruskal-Wallis test (P)	Dunn test (P)	
T30min	lStapicalmean (%)	51.3 (20.6–57.1)	**0.04979**	lStapicalmean - lStmidmean	0.068
	lStbasalmean (%)	16.9 (−20.7–43.5)		lStapicalmean - lStbasalmean	0.102
	lStmidmean (%)	12.2 (1.9–45.2)		lStmidmean - lStbasalmean	1.000
T2h	lStapicalmean (%)	42.7 (35.9–63.5)	**0.009**	lStapicalmean - lStmidmean	**0.027**
	lStbasalmean (%)	5.75 (−7.1–23.6)		lStapicalmean - lStbasalmean	**0.014**
	lStmidmean (%)	9.6 (3.8–29.8)		lStmidmean - lStbasalmean	0.724
T4h	lStapicalmean (%)	32.8 (19.1–40)	0.160	lStapicalmean - lStmidmean	
	lStbasalmean (%)	11.3 (−4.6–46.1)		lStapicalmean - lStbasalmean	
	lStmidmean (%)	22.3 (6.2–35.6)		lStmidmean - lStbasalmean	
T6h	lStapicalmean (%)	17.1 (4.9–26.2)	0.692	lStapicalmean - lStmidmean	
	lStbasalmean (%)	19.1 (−2.4–40.3)		lStapicalmean - lStbasalmean	
	lStmidmean (%)	6.8 (−1.4–26.9)		lStmidmean - lStbasalmean	
T8h	lStapicalmean (%)	18.4 (4.8–40.9)	0.289	lStapicalmean - lStmidmean	
	lStbasalmean (%)	−11.1 (−19.5–20.2)		lStapicalmean - lStbasalmean	
	lStmidmean (%)	9.6 (2.7–20.7)		lStmidmean - lStbasalmean	
T24h	lStapicalmean (%)	−2.0 (−6.9–9.8)	0.403	lStapicalmean - lStmidmean	
	lStbasalmean (%)	1.15 (−15.2–30.6)		lStapicalmean - lStbasalmean	
	lStmidmean (%)	−5.8 (−15.8–8.5)		lStmidmean - lStbasalmean	

Speckle tracking imaging measurements were obtained at baseline (T0, before drug administration) and at 30 min (T30min), 2 h (T2h), 4 h (T4h), 6 h (T6h), 8 h (T8h), and 24 h (T24h) after intravenous injection of pimobendan in the five apparently healthy Beagle dogs included in the study. The absolute values of longitudinal strain were used for statistical analysis. Kruskal-Wallis tests were used to compare the variation from baseline between groups at each time point. When significant, post hoc pairwise comparisons were performed using Dunn’s test. *p*-values were adjusted using Holm’s method to correct for multiple comparisons. Statistically significant results (*p* < 0.05) are shown in bold. lStapicalmean, mean of peak systolic longitudinal strain of apical septal and apical lateral segments averaged from three apical views; lStbasalmean, mean of peak systolic longitudinal strain of basal septal and basal lateral segments averaged from three apical views; lStmidmean, mean of peak systolic longitudinal strain of mid septal and mid lateral segments averaged from three apical views.

#### Left atrial function

A rapid increase in LA contractile function was observed following pimobendan administration, as evidenced by a significant increase in LAEF between T30min and T2h and in LAFS between T30min and T6h, along with a significant decrease in LADVol between T30min and T4h (Supplementary Table H and Supplementary Figure J).

### Parameters associated with stroke volume

The increase in SV appeared to be more associated with the enhancement of absolute values of lStglobalmean than rStglobal or cStglobal and particularly with the apical component (lStapicalmean) of longitudinal strain (Table 8). Univariate analyses indicated a relationship between plasma concentrations of ODMP and SV; however, this association was not retained in the multivariable analysis.

**Table 8:** Association between stroke volume and plasma drug concentrations or left ventricular absolute systolic strain values.

Dependent variable	Independent variable	Estimate	95% confidence interval	VIF
Stroke volume (mL)	rStglobal	0.05	−0.02; 0.10	2.27
	cStglobal	0.14	−0.18; 0.41	2.40
	lStglobalmean	**0.79**	**0.41; 1.17**	3.15
Stroke volume (mL)	lStmidmean	0.06	−0.28; 0.46	1.34
	lStbasalmean	0.34	−0.03; 0.68	2.86
	lStapicalmean	**0.55**	**0.35; 0.74**	2.36
Stroke volume (mL)	Pimobendan	0.05	−0.005; 0.09	
Stroke volume (mL)	ODMP	**0.19**	**0.02; 0.35**	
Stroke volume (mL)	Pimobendan	−0.10	−0.28; 0.08	13.16
	ODMP	0.50	−0.08; 1.08	13.16

All systolic strain values used are absolute values. This table summarizes the results of four linear mixed models. In each model, the dog’s name was included as a random effect. Estimates are presented with their 95% confidence intervals. Significant results are highlighted in bold. The high VIF values observed for pimobendan and ODMP in the multivariable model reflect their strong pharmacokinetic correlation (*r* = 0.95), which likely explains why neither compound reached statistical significance when combined, and the results of this model should therefore be interpreted with caution. lStapicalmean, mean longitudinal strain of the apical septal and apical lateral segments averaged from three apical views; lStbasalmean, mean longitudinal strain of the basal septal and basal lateral segments averaged from three apical views; cStglobal, mean of peak circumferential strain assessed at the endocardial, mid-myocardial and epicardial levels; lStglobalmean, mean of the peak longitudinal strain of the six left ventricular segments from the left apical four-, five- and two-chamber views; lStmidmean, mean longitudinal strain of the mid septal and mid lateral segments averaged from three apical views; ODMP, O-desmethylpimobendan; rStglobal, the mean of the rSt of the 6 LV segments, VIF, variance inflation factor.

### Diastolic left heart function

An increase in the E wave was observed between T30min and T8h. No statistically significant changes in E′l or E ′s were observed. Although a non-significant trend toward shortening of the heart rate-corrected IVRT (IVRT/RR) was noted, results were highly variable across individuals (Supplementary Tables I,J and Supplementary Figures K,L).

### Heart rate and arterial pressure

One dog developed a self-limiting episode of sinus tachycardia (heart rate >160 bpm) following pimobendan administration, from a few minutes after injection to approximately 1.5 h after (pink curve, Supplementary Figure M). Variations in blood pressure were not significant, and substantial interindividual variability was noted (Supplementary Table K and Supplementary Figure M).

## Discussion

Pimobendan administration was associated with increased LV contractility, as reflected by enhanced radial thickening for at least 6 h post-injection, circumferential shortening for at least 4 h, and longitudinal shortening for at least 8 h. However, substantial inter-individual variability was observed in these durations. Interestingly, in one dog, pimobendan appeared to resynchronize a dyskinetic myocardial segment at baseline, which subsequently normalized following drug administration. Although this observation is anecdotal, it raises the intriguing possibility that pimobendan may, in some cases, restore synchronous contraction of mildly dyskinetic segments, a finding that would warrant confirmation in larger studies, designed specifically to address this question. From a pharmacodynamic perspective, the effect of pimobendan on SV appeared to be more closely associated with plasma concentrations of ODMP than with pimobendan itself. Among all deformation indices, longitudinal strain, particularly at the apical level, showed the strongest association with SV. Finally, we demonstrated that a systolic longitudinal strain gradient exists from the base to the apex and that pimobendan administration enhances longitudinal contractility, particularly in the apical region. This suggests that the drug may have a relatively greater effect at the apex, which is consistent with our model indicating that apical longitudinal strain was the segment most closely associated with the increase in SV.

Following intravenous administration, pimobendan plasma concentrations increased very rapidly, with peak values already reached at the earliest sampling point (T30min). The true peak likely occurred earlier than 30 min, but it could not be captured given the sampling schedule. The plasma concentration of ODMP also increased very rapidly, but it remained detectable in the bloodstream for a longer period. As illustrated in Supplementary Figure A, inter-individual variability was visibly greater for ODMP than for pimobendan. The time course of both pimobendan and ODMP concentrations in our study was broadly consistent with previously published pharmacokinetic data (20, 39). However, by presenting individual concentration–time profiles rather than aggregated values alone, our study provides a clearer depiction of inter-individual variability. The greater dispersion in ODMP concentrations is pharmacokinetically plausible. Indeed, ODMP exposure reflects both the formation of the metabolite through hepatic oxidative demethylation and its subsequent clearance through phase II conjugation and biliary excretion, whereas the concentration–time profile of intravenously administered pimobendan mainly depends on distribution and high hepatic blood-flow-dependent clearance (40). Because pimobendan exhibits a high systemic clearance, its elimination is largely flow-limited, meaning that small differences in hepatic blood flow can directly influence the rate at which the parent compound is removed from the circulation (41). Small differences between dogs in hepatic enzyme activity potentially induced by cytochrome P450 polymorphism (42), hepatic blood flow (potentially influenced by the individual hemodynamic response to pimobendan), protein binding, or conjugating capacity are therefore likely to have a larger impact on ODMP concentrations than on the parent drug concentrations, resulting in a more heterogeneous metabolite profile across individuals. However, the unusual biphasic pattern observed in the green profile (Supplementary Figure A) is more likely attributable to an analytical or sampling artifact than to true physiological or pharmacokinetic variability.

To our knowledge, this is the first study to describe the effects of injectable pimobendan on LV systolic function over a 24-h period, using a longitudinal design with repeated echocardiographic assessments during the first 8 h after administration. The majority of previous investigations evaluating intravenous pimobendan in dogs have focused on very short time frames, typically within the first hour, and only a few extended their observations up to 2 h post-administration (17, 18, 23–25, 43). Such short-duration studies do not allow for characterization of the temporal profile of the drug’s inotropic effect. In contrast, the extended monitoring implemented in our study provides a clearer picture of the onset, magnitude, and duration of pimobendan-induced changes in myocardial contractility. In addition, our study benefits from being conducted in conscious Beagle dogs. Previous investigations assessing the cardiovascular effects of injectable pimobendan were performed under anesthesia, a condition that can influence cardiac contractility, vascular tone, and hemodynamic responses (44–46). Therefore, the use of anesthetized animals in earlier studies introduces potential confounding effects that may obscure the true inotropic profile of pimobendan. By evaluating the drug in awake dogs, our study provides a more physiologically relevant assessment of its impact on LV function.

Our results show that intravenous pimobendan significantly increases both radial and global conventional echocardiographic indices of LV contractility. This positive inotropic effect remained significant for 6 to 8 h after administration, depending on the parameter assessed, with the greatest changes generally observed at the 2-h time point. Because echocardiography was not performed continuously, the precise time of the peak effect could not be determined. Fractional shortening and LVEF both increased significantly for up to at least 6 h post-administration, with peak changes of approximately +20% at 2 h. A comparable pattern was observed for LVFAC, with a + 23% increase at 2 h and a significant enhancement that persisted for at least 8 h. Finally, SV increased by approximately +50% at 2 h and remained significantly elevated for at least 8 h. The overall inotropic effect of injectable pimobendan has been well described in previous studies, which consistently reported increases in myocardial contractility, CO, and SV during the first 1 to 2 h following administration (17–20). These effects have also been documented in anesthetized dogs, where the magnitude of the SV increase reported in previous studies is comparable to that observed in our cohort (17). Surprisingly, very few studies have reported short-term echocardiographic changes following intravenous pimobendan administration. In one of these investigations, and contrary to what would be expected from the known pharmacological profile of the drug, intravenous pimobendan did not produce a statistically significant increase in LVEF or LVFS during the first hour after administration, although a mild numerical rise in both indices was observed on average (23). Another study reported similar findings, with only modest changes in LVFS and LVEF 15 min after intravenous pimobendan administration (24). These observations were most likely influenced by the depressant effects of general anesthesia on cardiac function.

Interestingly, in our study, the changes in LV volumes and LVEF were similar when assessed from the right and left parasternal views, which is consistent with previous evidence showing that these measurements can be used interchangeably between these acoustic windows in healthy conscious dogs (47).

In the current study, we observed a significant increase in all three tested components of LV systolic strain, i.e., radial, circumferential, and longitudinal, following intravenous pimobendan administration, with maximal effects occurring approximately 2 h after drug injection. This finding was supported by the pharmacodynamic Tmax estimated from continuous spline models in the sensitivity analysis, which consistently identified a peak response between 166 and 195 min across all strain parameters (Supplementary Sensitivity Analysis). The magnitude of this response, however, differed between components. Systolic rSt showed the most pronounced change, increasing by approximately 70% compared with baseline, whereas circumferential and longitudinal strain increased more moderately, by approximately 20%. To the authors’ knowledge, only one study has previously measured LV systolic strain in dogs following pimobendan administration, and its findings are broadly consistent with ours. In that study, which evaluated strain in anesthetized dogs after pimobendan injection, anesthesia led to a reduction in radial, circumferential, and longitudinal strain values; however, this decrease was attenuated in dogs receiving pimobendan, at least during the first hour post-administration. The protective effect was most evident for rSt, with a less pronounced but still observable preservation of the cSt and lSt components (23). Taken together, these findings suggest that pimobendan exerts a more pronounced effect on systolic radial myocardial thickening than on the other components of LV systolic deformation. Nevertheless, this radial response does not appear to be the primary driver of the drug’s hemodynamic effects, particularly regarding the increase in SV. In our study, SV changes were more closely associated, at least from a statistical standpoint, with enhancements in longitudinal contractility and especially with the apical component of longitudinal strain. The apical segment was indeed the most affected when compared with the basal and midventricular segments, indicating that regional improvements in apical longitudinal shortening were statistically associated with the SV augmentation observed after intravenous pimobendan administration, although a causal relationship cannot be established from this observational design.

The canine LV myocardium is organized into three distinct muscular layers with specific myofiber orientations (48). The superficial (subepicardial) and inner (subendocardial) layers are predominantly composed of longitudinally oriented fibers, whereas the middle layer consists of a thick band of circumferentially oriented cardiomyocytes, which represents the largest myocardial mass and contributes most substantially to global LV systolic performance. Radial strain, similar to circumferential strain, is strongly influenced by afterload (49), and therefore reflects load-dependent myocardial wall thickening rather than true cavity shortening. Consequently, marked increases in rSt are expected under the influence of an inodilator such as pimobendan, since the drug simultaneously enhances contractility and reduces afterload through vasodilation. Thus, while part of the increase in systolic rSt observed in our study may reflect genuine improvements in systolic wall thickening, a substantial proportion is also likely explained by afterload-dependent mechanisms secondary to pimobendan-induced vasodilation. Furthermore, pimobendan reduces preload through venodilation, which may also influence strain measurements independently of its inotropic effect, as preload reduction alters both myocardial fiber stretch and ventricular geometry at end-diastole and has been shown to significantly affect strain values in all directions in animal models (50). Formally dissociating the inotropic contribution from these combined loading effects would require invasive pressure-volume analysis, which was beyond the scope of the present study.

We identified a consistent systolic longitudinal strain gradient along the LV axis, with higher values in the apical segment compared to the mid and basal regions (Figure 6b and Supplementary Figure H). Similar findings have been described in both humans (51) and dogs (10), in which LV longitudinal strain values progressively increase from the base to the apex. This gradient, with a more pronounced increase in apical contraction, is consistent with the fact that the apical region contributes more substantially to SV than the mid or basal segments, as previously demonstrated in humans (51). In our study, this gradient was present at all study time points, both before and after pimobendan administration. However, pimobendan accentuated the difference between the apical segment and the other regions, suggesting that its effect on longitudinal contractility is particularly pronounced in the apical myocardium. It should be acknowledged that apical segments are known to be particularly susceptible to acquisition-related artifacts in STE, notably apical foreshortening (52, 53), which can lead to the overestimation of apical strain values, and which may have partially influenced the magnitude of the apical strain measurements reported in the present study. However, in our study, all measurements were performed under standardized conditions by the same operator using the same equipment, and the within-subject repeated-measures design means that any systematic acquisition bias would have affected all time points equally. The observed temporal variation in apical strain (including its return toward baseline values at T24h) is therefore unlikely to be primarily explained by measurement artifacts and is more consistent with a genuine pharmacodynamic effect.

When analyzing three of the components of LV contractility, our results demonstrated that longitudinal LV contraction, as assessed by lSt, showed the strongest association with SV. To the authors’ knowledge, this relationship has not been previously described in dogs. In humans, systolic longitudinal shortening of the LV is known to account for the majority of SV generation (54). This motion predominantly results from the systolic displacement of the atrioventricular plane toward the apex, which contributes approximately 60% of the SV in both healthy individuals and patients with chronic myocardial infarction, where longitudinal shortening remains the principal parameter associated with SV (54–56). The remaining 40% of SV arises from the circumferential contraction of the thick mid-myocardial layer, which is composed of circumferentially oriented cardiomyocytes, with the interventricular septum and lateral wall contributing approximately 8 and 30%, respectively, to the SV (57). Another study in humans suggested that the contribution of longitudinal shortening to SV may be even greater - approximately 75% compared to 25% from short-axis shortening- regardless of age or body size (58). It should be noted, however, that the canine myocardium contains a higher proportion of circumferentially oriented fibers relative to longitudinally oriented fibers compared to the human heart, with a reported ratio of approximately 10:1 at the mid-myocardial level (59), which may limit the direct extrapolation of these human data to dogs. Nonetheless, the present observational findings in dogs are consistent with an important functional role for longitudinal deformation in SV generation, and the question of the relative contribution of each strain component to SV in the canine species warrants further dedicated investigation. Thus, the strong statistical association we observed between SV and the longitudinal strain component is likely not exclusively attributable to the effect of pimobendan, as longitudinal shortening is already the principal determinant of SV under normal physiological conditions. To clearly delineate the specific pharmacodynamic contribution of pimobendan to this relationship, the inclusion of a control group would have been necessary to allow direct comparison between treated and untreated animals.

In cardiac disease, myocardial dysfunction is often heterogeneous, and distinct patterns of regional impairment or failure phenotypes may emerge. This has been particularly well described in dogs with dilated cardiomyopathy, in which all strain components tend to be reduced, with a disproportionately marked decrease in radial strain (10, 11, 60). In addition, regional differences in contractility have been reported, with a more pronounced impairment of apical function compared with mid- and basal segments, particularly with regard to longitudinal strain (10). As discussed above, our results suggest that the effects of pimobendan are not limited to a strong enhancement of radial strain but also include a marked increase in apical longitudinal contraction. We have also highlighted that longitudinal deformation - particularly at the apical level - is of major hemodynamic importance, as it plays a key role in determining SV. Taken together, these findings help explain why pimobendan is clinically effective in dogs with dilated cardiomyopathy, a condition in which both radial thickening and apical longitudinal shortening are markedly impaired (12, 16, 61). However, it should be emphasized that the present study was conducted in healthy Beagle dogs and extrapolation of these findings to dogs with naturally occurring DCM should be made with caution. Whether the strain enhancement profile observed here would be replicated in a structurally and functionally remodeled myocardium remains to be determined in future studies.

In the present study, pimobendan injection also significantly improved LA contractile function. A previous study evaluated LA function before and 15 min after pimobendan injection in six healthy, anesthetized Beagles using both conventional echocardiography and 2D-STE (24). The increase in LAFS and LAEF did not reach significance 15 min after pimobendan injection, whereas LA booster pump function parameters (active LAEF and longitudinal strain) did. In our study, LAFS and LAEF were increased between T30min and T6h and between T30min and T2h, respectively. This difference between the two studies could be explained in part by the conscious *versus* anesthetized status of the dogs, and in part by the fact that the previous study only reassessed LA function 15 min after pimobendan injection and not at later time points. However, the evidence regarding pimobendan’s effects on LA function is not uniform across studies. Sarcinella et al. evaluated LA function before and up to 6 months after initiation of oral pimobendan in dogs with preclinical MMVD and found no significant change in any LA variable, despite a concurrent improvement in LV systolic function (62). Similarly, Abbott-Johnson et al. conducted a randomized placebo-controlled crossover study in seven Beagles with tachycardia-induced DCM and found no significant difference in LA emptying fraction, LA functional index, LA:Ao ratio, or mitral A wave velocity between pimobendan and placebo 3 h after oral administration (15). Interestingly, these authors observed significant reductions in LA minimum and maximum volumes following pimobendan treatment compared to baseline, a finding they cautiously interpreted as possibly reflecting direct atrial inotropism but acknowledged that improved ventricular lusitropic function, which was also documented in their study, could equally account for this change. These discrepancies across studies likely reflect the difficulty of evaluating atrial function in isolation from ventricular function and significant differences in study design, disease status, route and duration of drug administration, and loading conditions. The effect of pimobendan on LA function is complex: in particular, its vasodilatory properties may decrease LA preload and thereby attenuate any intrinsic increase in atrial contractility through a Frank-Starling mechanism. Moreover, in the context of mitral regurgitation, this vasodilation reduces LV afterload and, consequently, mitral regurgitant volume, thereby affecting LA function. Therefore, the observed increases in LAFS and LAEF in our study should be interpreted cautiously, as they likely reflect a combination of mechanisms rather than a purely direct atrial inotropic effect. Specifically, improved ventricular lusitropy reducing LA afterload, and altered loading conditions secondary to enhanced LV systolic performance may have all contributed to the observed changes. Finally, the absence of LA strain data in the present study represents a limitation, as these indices would have provided a more comprehensive characterization of the atrial response to pimobendan.

The observed increase in E-wave velocity warrants careful mechanistic interpretation. Peak E-wave velocity is determined by both the rate of active LV relaxation and the transmitral pressure gradient, the latter of which depends on LA pressure and, consequently, preload. Because pimobendan simultaneously enhances LV lusitropy and reduces preload through venodilation, these two effects exert opposing influences on E-wave velocity (15, 62). The fact that E-wave velocity increased despite a probable reduction in LA preload suggests that the lusitropic effect predominated over the preload-reducing effect in determining the transmitral filling pattern. However, this interpretation should be tempered by several observations. First, E′l and E’s did not change significantly, suggesting that the increase in E-wave velocity may not solely reflect enhanced intrinsic myocardial lusitropy. Second, the heart rate-corrected IVRT showed a non-significant trend toward shortening, but the results were highly variable across individuals (Supplementary Figure L), precluding any definitive conclusion regarding a lusitropic effect. Finally, E deceleration time was not measured in the present study, which represents a limitation, as this parameter would have provided additional mechanistic insight into whether the observed increase in E-wave velocity reflects a relaxation-driven or a pressure-driven filling pattern.

This study has several limitations that should be acknowledged. First, the number of dogs included was relatively small. Although this sample size was sufficient to detect significant increases in the assessed echocardiographic parameters, a larger cohort would likely provide a more refined characterization of inter-individual variability. Second, one limitation of our study is that only male dogs were included. This decision was made to ensure a more homogeneous study population. Third, the absence of a control group represents a major limitation of this study and restricts the strength of causal inference regarding the effects of pimobendan on SV. Without an untreated or placebo-controlled comparison group, it cannot be excluded that part of the observed changes may be related to factors other than the drug itself, such as operator learning effects, circadian variations in cardiac function, or the intrinsic variability of speckle-tracking echocardiography measurements. In addition, the potential influence of the experimental conditions, including handling, restraint, and arterial catheterization, cannot be disregarded. This design was deliberately chosen in order to comply with the principles of reduction within the 3Rs framework for animal research by limiting the number of animals exposed to invasive procedures. However, we acknowledge that a placebo-controlled crossover design would have strengthened the robustness of the conclusions and allowed for a more rigorous assessment of the specific contribution of pimobendan. The within-subject design partially mitigates this limitation, as each dog served as its own control. The availability of baseline measurements prior to pimobendan administration, together with 24-h follow-up measurements after the expected dissipation of the drug’s effects, showed that most variables returned close to their initial values. While this supports the internal consistency of the observations, it does not fully compensate for the absence of an independent control group. Fourth, although anesthetic agents were used for catheter placement and could have theoretically influenced baseline measurements, particularly by transiently reducing cardiac function, this appears unlikely. A sufficiently long recovery period was allowed to ensure the clearance of alfaxalone and midazolam before baseline data collection, and both drugs are known to have a limited or no impact on the echocardiographic indices (63–65). Fifth, the most important limitation of this study is that it was conducted in healthy young dogs rather than in animals with cardiac disease. It is entirely possible, and even likely, that the effects of pimobendan on a normal heart do not fully reflect its impact on a diseased myocardium. Nevertheless, the use of healthy dogs was justified on ethical grounds, as the study protocol was relatively demanding and required multiple sequential echocardiographic examinations that could have induced unnecessary stress or clinical deterioration in dogs with a cardiac pathology. Finally, a limitation inherent to the sampling schedule of this study is that the first blood collection was performed at 30 min post-injection, at which point pimobendan plasma concentrations had already reached the maximal concentration. This was not an initial objective of the study, which was primarily designed to characterize the temporal profile of pimobendan’s effects on myocardial deformation rather than to perform formal pharmacokinetic–pharmacodynamic modeling. Nonetheless, this design precluded the characterization of the ascending phase of the concentration–time profile and, consequently, prevented the construction of formal compartmental PK-PD models. This limitation is particularly relevant given the stronger apparent pharmacodynamic association between SV and ODMP concentrations compared with the parent compound. Future studies incorporating a denser early sampling strategy would allow formal compartmental PK-PD modeling and would help clarify whether the stronger apparent pharmacodynamic signal associated with ODMP reflects genuine differences in pharmacological potency or is an artifact of the sampling design.

## Conclusion

Our findings indicate that a single intravenous bolus of pimobendan produces a measurable and clinically relevant enhancement of left-heart function, lasting approximately 6 to 8 h, with the peak effect most likely occurring approximately 2 h after administration. Using STE, we showed that pimobendan markedly increases systolic radial LV strain and enhances systolic longitudinal LV deformation, particularly in the apical segments. This apical longitudinal component showed the strongest statistical association with the observed increase in SV. Whether this reflects a mechanistic contribution of apical longitudinal shortening to pimobendan’s inotropic action cannot be established from this observational study. Overall, this study provides novel insights into the temporal and regional mechanics underlying pimobendan’s inotropic effects in awake dogs. As this study was exploratory in nature, the findings reported here will require confirmation in future studies. Such studies would ideally include larger cohorts and, most importantly, animals with cardiac disease, in order to determine whether the effects of pimobendan on myocardial mechanics observed in healthy dogs are preserved, amplified, or attenuated in a diseased myocardium, which represents the primary clinical target of this drug. Future investigations should also include females, other breeds, and different age groups to fully assess the generalizability of the present findings.

## Data Availability

The raw data supporting the conclusions of this article will be made available by the authors, without undue reservation.

## Glossary

2D: Two-dimensional
A′l: Late diastolic annular velocity at the lateral mitral annulus
A′S: Late diastolic annular velocity at the septal mitral annulus
Ao: Aortic diameter
AoD: Aortic diameter
CO: Cardiac output
cSt: Systolic circumferential strain
cStendo: Peak circumferential strain at the endocardial level
cStmid: Peak circumferential strain at the mid-myocardial level
cStepi: Peak circumferential strain at the epicardial level
cStglobal: Mean of peak cStendo, cStmid and cStepi
DAP: Diastolic arterial pressure
DCM: Dilated cardiomyopathy
E: Peak early mitral inflow velocity
E′l: Early diastolic annular velocity at the lateral mitral annulus
E′s: Early diastolic annular velocity at the septal mitral annulus
EPSS: E-point-to-septal separation
IVRT: Isovolumetric relaxation time
LA: Left atrium
LADVol: Left atrial diastolic volume
LAEF: Left atrial ejection fraction
LAFS: Left atrial fractional shortening
LAmax: Maximal left atrial diameter
LAmin: Minimal left atrial diameter
LASVol: Left atrial systolic volume
lSt: Systolic longitudinal strain
lStglobal4ch: Mean of peak lSt of the six left ventricular segments from the left apical four-chamber view
lStglobal5ch: Mean of peak lSt of the six left ventricular segments from the left apical five-chamber view
lStglobal2ch: Mean of peak lSt of the six left ventricular segments from the left apical two-chamber view
lStglobalmean: Mean of lStglobal4ch, lStglobal5ch and lStglobal2ch
lStbasal: Peak lSt averaged from the basal septal and basal lateral segments;
lStmid: Peak lSt averaged from the mid septal and mid lateral segments
lStapical: Peak lSt averaged from the apical septal and apical lateral segments
lStbasalmean: Mean of lStbasal from the left apical two-, four-, and five-chamber views
lStmidmean: Mean of lStmid from the left apical two-, four-, and five-chamber views
lStapicalmean: Mean of lStapical from the left apical two-, four-, and five-chamber views
LV: Left ventricle
LVFAC: Left ventricular fractional area change
LVFS: Left ventricular fractional shortening
LVEF: Left ventricular ejection fraction
LVIAd: Left ventricular internal area at end-diastole
LVIAs: Left ventricular internal area at end-systole
LVIDd: Left ventricular internal diameter at end-diastole
LVIDs: Left ventricular internal diameter at end-systole
LVDVol: Left ventricular diastolic volume
LVSVol: Left ventricular systolic volume
MAP: Mean arterial pressure
MMVD: Myxomatous mitral valve disease
ODMP: O-desmethylpimobendan
PK-PD: Pharmacokinetic-pharmacodynamic
RR: RR interval
rSt: Systolic radial strain
rStglobal: Mean of peak rSt of the six left ventricular segments
S′l: Systolic annular velocity at the lateral mitral annulus
S′s: Systolic annular velocity at the septal mitral annulus
SAP: Systolic arterial pressure
STE: Speckle-tracking echocardiography
SV: Stroke volume
TDI: Tissue Doppler imaging
VIF: Variance inflation factor
VTIAo: Aortic velocity time integral
