# Supplementary material for: Characterization of global and regional left heart function using advanced echocardiography following intravenous administration of pimobendan in awake dogs
Source: Front Vet Sci. 2026 Jul 3;13:1842861. doi: 10.3389/fvets.2026.1842861 (PMC13375486; doi:10.3389/fvets.2026.1842861)
Supplement: Supplementary file 1 [file Data_Sheet_1.docx]

**Characterization of global and regional left heart function using advanced echocardiography following intravenous administration of pimobendan in awake dogs**

Pierre Foulex* ^a,b,c^, Valérie Chetboul*^d,e^, Emilie Moisset^f^, Fany Roncin ^f,g^, Alona Dougère^h^, Guillaume Noël^h^, Emilie Tréhiou^i^, Céline Pouzot-Nevoret^g^, Valentin Bondoux^i^, Mathieu Magnin^f,g **^

**Affiliations**

1. Clinique vétérinaire Boulogne Roland-Garros, Boulogne-Billancourt, France
2. Sudvetia, Aix-en-Provence, France
3. Veranex France, Paris, France
4. Ecole Nationale Vétérinaire d’Alfort, CHUV-AC, Maisons-Alfort, France
5. Université Paris Est Créteil, INSERM, IMRB, Créteil, France
6. Université de Lyon, Vetagro Sup, Unité de Physiologie, Pharmacodynamie et Thérapeutique, 1 avenue Bourgelat F-69280 Marcy l’Etoile, France
7. Université de Lyon, APCSe Agressions Pulmonaires et Circulatoires dans le Sepsis (RSNR 201622884J), VetAgro Sup, 1 avenue Bourgelat F-69280 Marcy l’Etoile, France
8. Université de Lyon, VetAgro Sup, Biovivo/Institut Claude Bourgelat, 1 avenue Bourgelat F-69280 Marcy l’Etoile, France
9. Université de Lyon, VetAgro Sup, Service de cardiologie, 1 avenue Bourgelat F-69280 Marcy l’Etoile, France
10. Université de Lyon, VetAgro Sup, Unité de Pharmacie et de Toxicologie, 1 avenue Bourgelat F-69280 Marcy l’Etoile, France

* These two authors contributed equally to this work

** corresponding author: Mathieu Magnin, [mathieu.magnin@vetagro-sup.fr](mailto:mathieu.magnin@vetagro-sup.fr), VetAgro Sup, 1 avenue Bourgelat, 69280 Marcy L’Etoile, France. ORCID: 0000-0003-0898-5425

**Supplementary Method A. Detailed experimental protocol and plasma quantification of pimobendan and O-desmethylpimobendan**

1. Synopsis

A clinical examination was performed, followed by the insertion of a peripheral venous catheter into the cephalic vein. The dogs were then anesthetized using alfaxalone (2 mg/kg IV), butorphanol (0.2 mg/kg IV), and midazolam (0.2 mg/kg IV). A femoral arterial catheter was placed percutaneously using the Seldinger technique. Upon recovery, meloxicam (0.05 mg/kg SC) was administered for analgesia. Two hours after anesthetic administration, a bolus of pimobendan (0.15 mg/kg IV) was administered.

The dogs were then monitored for a period of 8 hours. During this time, arterial blood pressure, including systolic (SAP), mean (MAP), and diastolic (DAP) pressures, and electrocardiographic parameters were continuously recorded (Carescape B650, GE Healthcare, United Kingdom). Clinical and echocardiographic examinations were performed at baseline (T0), and then, 30 minutes (T30min), 2 hours (T2h), 4 hours (T4h), 6 hours (T6h), 8 hours (T8h) and 24 hours (T24h) after pimobendan injection.

Three milliliters of arterial blood were collected from the catheter at T0, 10 minutes, 20 minutes, 30 minutes, 1 hour, 2 hours, 4 hours, 6 hours, 8 hours and 24 hours to measure plasma concentrations of pimobendan and its metabolite, O-desmethylpimobendan (ODMP) using Liquid Chromatography-Tandem Mass Spectrometry (see details below).

During the 8 hours following pimobendan injection, the dogs were housed in individual cages. They were walked for 15 minutes at T4h, and throughout this period they had access to toys, water, and food. At T8h, the catheters were removed, and the dogs were then returned to their group cages until the next evaluation. At T24h, a follow-up clinical and echocardiographic examination was performed, and a blood sample was collected from the jugular vein.

2. Plasma quantification of pimobendan and O-Desmethylpimobendan

Blood samples were drawn into tubes coated with lithium heparin and centrifuged at 5,000 × g at 4°C for 10 minutes to separate the plasma within an hour of collection. The isolated plasma was then stored at −20°C for further analysis. Before analysis, the plasma samples were thawed at room temperature.

Pimobendan and ODMP concentrations were assessed using Liquid Chromatography - Tandem Mass Spectrometry (LC-MS/MS). The LC-MS/MS system consisted of an Agilent 1260 Infinity II coupled with a 6470A triple quadrupole mass spectrometer (Agilent technologies, France) including an electrospray ionization source (ESI). The chromatographic separation was performed using a Restek Raptor Biphenyl column (50 x 2.1 mm, 2.7 μm) connected to a guard column (5 x 2.1 mm, 2.7 μm). The mobile phase consisted in 0.2% formic acid in water (A) and methanol (B). A flow rate of 0.5 mL/min was maintained, and the analytes were eluted using the following gradient: 0 min, 10% B; 0.5 min, 10% B; 1.5 min, 90% B; 3 min, 90% B; 4.5 min, 10% B. The ESI system was operated in the positive mode with the following fixed settings: capillary voltage 3 kV; gas temperature 200°C at a gas flow of 8 L/min of nitrogen; Sheath gas temperature 300°C at a gas flow of 10 L/min nitrogen, nebulizer 30 psi. Dwell time was set to 200 ms for all compounds. The mass-to-charge ratios were 335.2/319.1 and 335.2/276 for pimobendan, 321.1/305 and 321.1/236 for ODMP and 338.2/322.1 for pimobendan-D_3_. Internal standard (IS) solution was prepared at 1 µg/g for pimobendan-D_3_ in acetonitrile. A plasma sample aliquot of 100 µL was mixed with 5 µL of internal standard solution and 395 µL of acetonitrile. The mixture was vortexed and centrifuged at 10,000 rpm for 5 min. Then, the supernatant was transferred to a sample vial for analysis (V_inj_: 5 µL). Fortified samples with pimobendan and ODMP were prepared using the procedure described above for calibration. The linearity range was 10-150 ng/mL. The lower limit of quantification (LLOQ) was 10 ng/mL for both pimobendan and ODMP. Method precision was assessed at four concentration levels across the calibration range. Intra-day precision was 6% and 9% for pimobendan and ODMP, respectively, and inter-day precision was 14% and 15%, respectively, all below the accepted threshold of 20%. Calibration standards were prepared in plasma matrix to account for matrix effects and ensure adequate analyte recovery. Pimobendan-D_3_, ODMP and pimobendan solution standard were purchased from CIL (France). The LC-MS grade methanol and acetonitrile were obtained from Fisher Scientific (France). Formic acid (> 98%) was obtained from Sigma-Aldrich Chemie (France).

**Supplementary Method B. Detailed protocol for transthoracic echocardiographic acquisition and analysis including conventional two-dimensional (2D) and M-mode measurements, Doppler-derived cardiac output estimation, tissue Doppler imaging (TDI), and speckle-tracking echocardiography (STE) strain analysis**

Standard and advanced transthoracic echocardiography with continuous ECG monitoring was performed by the same investigator (PF) using a single ultrasonographic unit (Vivid IQ, General Electric Medical System, Waukesha, Wisc, USA) equipped with phased-array transducers, including 3Sc-RS (1.5–4.0 MHz) and 6S-RS (5.0–11.0 MHz) probes (General Electric Medical System, Waukesha, Wisc, USA). Echocardiographic examinations were performed in minimally restrained awake standing Beagle dogs as previously described and validated ^1^.

1. Conventional echocardiographic and Doppler examinations

1. Two-dimensional and M-mode measurements

Aortic (Ao) and left atrial (LA) diameters were measured at both end-diastole ^2^ and end-systole ^3^ using the two-dimensional (2D) method from the right parasternal transaortic short-axis view. Corresponding left atrium-to-aorta (LA:Ao) ratios were calculated. On the same view, LA internal diameters at end-diastole (LAmin) and end systole (LAmax) were obtained using the 2D-guided M-mode, to finally calculate LA fractional shortening (LAFS) using the formula: 100*(LAmax-LAmin)/LAmax ^4^.

Left ventricular linear measurements and notably end-diastolic and end-systolic LV internal diameters (LVIDd and LVIDs, respectively) were obtained using the 2D-guided M-mode from the right parasternal transventricular short-axis view ^5,6^, and the LV fractional shortening (LVFS) was calculated accordingly. E-point to septal separation (EPSS) was defined as the minimal diastolic distance between the anterior mitral valve leaflet and the interventricular septum and was measured using the 2D-guided M-mode from the right parasternal transmitral short-axis view. For each M-mode echocardiographic variable, a mean of 3 measurements was average and used for analysis, except for EPSS, for which only the minimal measured distance was retained.

Left ventricular volumes were measured at end-diastole (LVDVol) and end-systole (LVSVol) using Simpson’s method of discs by manually tracing the LV endocardial border at blood-tissue interface with exclusion of papillary muscles, in two image planes, from both the right parasternal long axis 4-chamber view and the left apical 4-chamber view ^7^. The LV ejection fraction (LVEF) was then calculated using the formula: 100*(LVDVol – LVSVol) / LVDVol) from both views.

Left atrial volume was calculated at both end-systole (LASVol) and end-diastole (LADVol) from the left apical 4-chamber view using the monoplane Simpson’s method of discs, as previously described and validated in dogs in the standing position^8^. The LA ejection fraction (LAEF) was then calculated using the formula: 100*(LASVol - LADVol)/ LASVol^9^.

End-diastolic and end-systolic LV internal area (LVIAd and LVIAs, respectively) were obtained by tracing the endocardial borders of the LV from the right parasternal transventricular short-axis view excluding the papillary muscles, and the LV fractional area change (LVFAC) was subsequently calculated using the formula: (LVIAd-LVIAs)/LVIAd ^7^.

1. Conventional Doppler examination and estimation of cardiac output

Peak early (E) and late (A) diastolic mitral flow velocities were assessed using pulsed-wave Doppler mode from the left apical 4-chamber view, and the mitral E:A ratio was subsequently calculated. The isovolumetric relaxation time (IVRT) was determined from the left apical 5-chamber view, defined as the interval between the end of aortic flow ejection and the onset of early mitral inflow.

The systemic (Qs) flow was indirectly assessed by combining 2D echocardiography and pulsed-wave Doppler mode^10^. The aortic diameter (AoD) was measured at end-systole by 2D method using the right parasternal 5-chamber view. The aortic flow velocity profile was assessed by pulsed-wave Doppler mode using the left apical 5-chamber view. The sample volume was positioned within the arterial flow stream just distal to the opened aortic valve, and particular effort was made to obtain the best alignment between arterial blood flow and the Doppler beam, without angle correction. Aortic velocity time integral (VTIAo) was manually traced from outer envelopes, and the SV was calculated using the following formula: SV (mL) = (π(AoD/2)^2^) x VTIAo^10^, with both AoD and VTIAo expressed in centimeters (cm). The cardiac output (CO) was then indirectly calculated as CO (mL/min) = SV x heart rate (HR, bpm).

2. Tissue Doppler Imaging

Peak early and late diastolic velocities as well as systolic velocities were measured at the lateral mitral annulus (E’l, A’l, and S’l, respectively) and also at the septal mitral annulus (E’s, A’s, and S’s, respectively), using pulsed-wave TDI from the left apical 4-chamber view with a sample volume of 3 mm^11^.

3. Myocardial Strain Imaging using speckle tracking echocardiography

Two-dimensional STE was used to assess LV regional and global deformation parameters, i.e., systolic strain (St) in longitudinal, radial and circumferential directions. Speckle tracking analysis was performed off-line by a single investigator (VC) with a high experience in STE, using the same software package (Echo Pac PC 6.3 software, GE Healthcare). The observer was blinded to the STE examination and particularly to the timing of the examination related to the pimobendan injection. Additionally, the observer was blinded to the TDI and conventional echocardiographic results while assessing the STE variables. The operator has over 20 years of experience in STE. Intra-observer reproducibility of STE-derived indices has been previously established by this same operator under comparable experimental conditions (awake standing dogs), with within- and between-day coefficients of variation ranging from 5.2 to 8.5%, supporting the reliability of the measurements reported in the present study^12^.

One single 2D cineloop with three consecutive cardiac cycles was acquired from each dog for STE examination from the following views: the right parasternal transventricular short-axis view for LV radial and circumferential strain, and the left apical 4-chamber, 5-chamber and 2-chamber views for LV longitudinal strain. For each loop, the cardiac cycle with the best visualization of endocardial and epicardial borders was chosen. Throughout the manuscript, all strain values are expressed as absolute values.

As previously described^13^, for systolic radial strain (rSt), the LV endocardial border was manually traced on the right parasternal transventricular short-axis view at end-systole while excluding papillary muscles. The computer software then automatically delimited the epicardial border, which was manually adjusted when necessary. Afterwards, the software automatically segmented the LV into six myocardial segments within the interventricular septum and the LV free wall (anteroseptal, anterior, lateral, posterior, inferior and septal) and selected suitable speckles for tracking. The software algorithm automatically searched for these speckles on a frame-by-frame basis using the sum of absolute difference algorithm. Six LV rSt profiles were then obtained, corresponding to the average values of each myocardial segment. The peak systolic rSt values were automatically assessed for each of the six curves. The global peak systolic LV rSt (rStglobal) was then obtained by averaging these six maximal rSt values. Myocardial synchrony was also assessed by the difference in timing of peak rSt from the earliest to latest segment^13^. HR was also calculated during each STE examination using concomitant ECG tracing.

The technique for systolic longitudinal strain (lSt) measurement was the same as that described for rSt, and peak systolic lSt was calculated from the left apical 4-, 5-, and 2-chambers views. For each view, peak lSt values were automatically assessed for each of the 6 LV segments (basal septal and basal lateral, mid septal and mid lateral, and apical septal and apical lateral). The global peak systolic LV lSt (lStglobal) was obtained by averaging 18 peak systolic lSt values, i.e., six from the left apical 4-chamber (lStglobal4ch), six from the left apical 5-chamber (lStglobal5ch), and six from the left apical 2-chamber view (lStglobal2ch). The mean value of lStglobal4ch, lStglobal5ch and lStglobal2ch was then calculated (lStglobalmean).

Furthermore, for each longitudinal view, the LV segments were grouped two-by-two according to LV level, and the lSt were averaged at each level:

- the basal LV: peak systolic lSt values were averaged from basal septal and basal lateral segments to obtain the peak systolic lSt of the basal LV (lStbasal);
- the mid LV: peak systolic lSt values were averaged from mid septal and mid lateral segments to obtain the peak systolic lSt of the mid LV (lStmid);
- the apical LV: peak systolic lSt values were averaged from apical septal and apical lateral segments to obtain the peak systolic lSt of the LV apex (lStapical).

These two-by-two associations of segments according to the LV level were performed on each view. For each lSt parameter, a suffix was added to indicate the echocardiographic view from which the measurement was obtained (2ch for the two-chamber view, 4ch for the four-chamber view, and 5ch for the five-chamber view)

Circumferential strain (cSt), defined as shortening of the myocardium along the LV circular perimeter in short axis^14^, was assessed from the right parasternal transventricular short-axis view using the same STE technique used for rSt and lSt. The values of peak systolic cSt were independently evaluated at endocardial (cStendo), mid-myocardial (cStmid), and epicardial (cStepi) levels to determine which LV myocardial layer was more responsive to pimobendan injection regarding circumferential contractility. The global cSt (cStglobal) was calculated by averaging cStendo, cStmid, and cStepi.

**Supplementary Table A. Evolution of plasma concentrations of pimobendan and O-desmethylpimobendan over time: results of linear mixed models.**

| Dependent variable | Independent Variable | Estimate | 95% confidence interval |
| --- | --- | --- | --- |
| Pimobendan (ng/mL) | **T10m** | **115.0** | **97.3; 132.5** |
|  | **T20m** | **83.4** | **65.75; 101.0** |
|  | **T30m** | **62.0** | **44.4; 79.6** |
|  | **T1h** | **25.1** | **7.5; 42.7** |
|  | T2h | 4.8 | -14.0; 23.6 |
|  | T4h | 1.5 | -16.2; 19.2 |
|  | T6h | 0.3 | -17.4; 17.9 |
|  | T8h | 0.1 | -17.5; 17.7 |
|  | T24h | 0.0 | -17.6; 17.6 |
| ODMP (ng/mL) | **T10m** | **11.7** | **6.5; 16.8** |
|  | **T20m** | **16.9** | **11.7; 22.0** |
|  | **T30m** | **18.5** | **13.3; 23.6** |
|  | **T1h** | **11.5** | **6.3; 16.6** |
|  | **T2h** | **7.9** | **2.3; 13.4** |
|  | **T4h** | **1.0** | **-4.1; 6.1** |
|  | T6h | 0.2 | -4.9; 5.3 |
|  | T8h | 0.0 | -5.1; 5.1 |
|  | T24h | 0.0 | -5.1; 5.1 |

Pimobendan (0.15 mg/kg IV) was administered intravenously to five healthy awake Beagle dogs immediately after the baseline (T0) measurements. The values at different time points were compared to those at T0 using a linear mixed model, with the dog’s name included as a random effect. In the table, the estimate represents the mean difference between each time point and T0. The estimate is presented along with its 95% confidence interval. Significant differences are indicated in bold. *ODMP: O-desmethylpimobendan.*

**
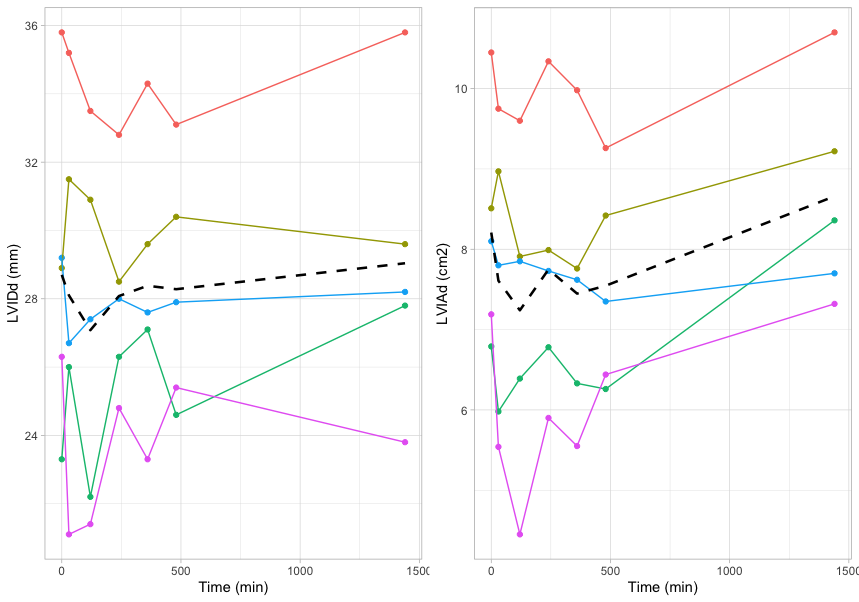
Supplementary Figure A. Time course of left ventricular internal diameter in diastole and left ventricular internal area in diastole following intravenous pimobendan administration in five healthy awake Beagle dogs.**

Pimobendan (0.15 mg/kg IV) was administered immediately after the baseline (T0) measurements. Each color represents an individual dog. The dashed black line represents the mean. *LVIDd: left ventricular internal diameter in diastole; LVAd: left ventricular internal area in diastole.*


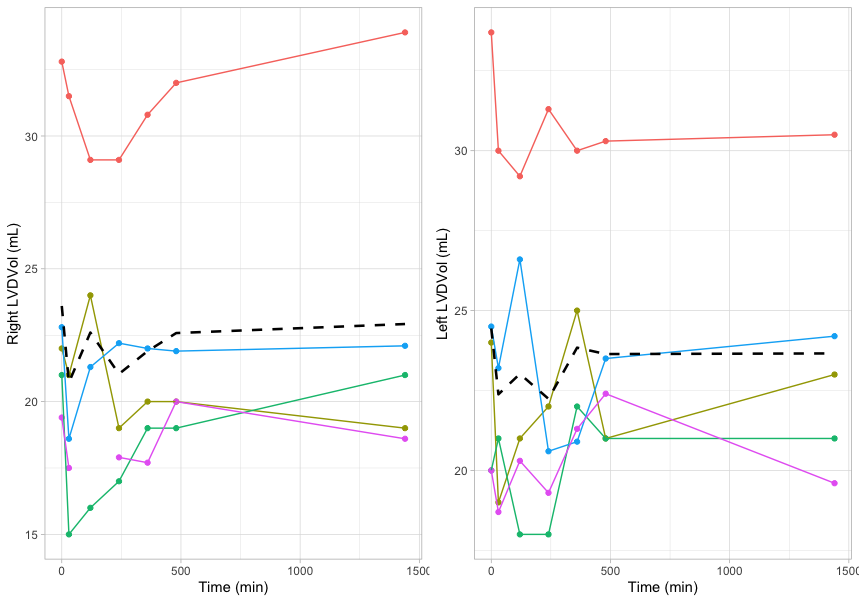
**Supplementary Figure B. Time course of right and left ventricular diastolic volumes following intravenous pimobendan administration in five healthy awake Beagle dogs**

Pimobendan (0.15 mg/kg IV) was administered immediately after the baseline (T0) measurements in five healthy awake Beagle dogs. Each color represents an individual dog. The dashed black line represents the mean. LVDVol: left ventricular diastolic volume; RVDVol: right ventricular diastolic volume.


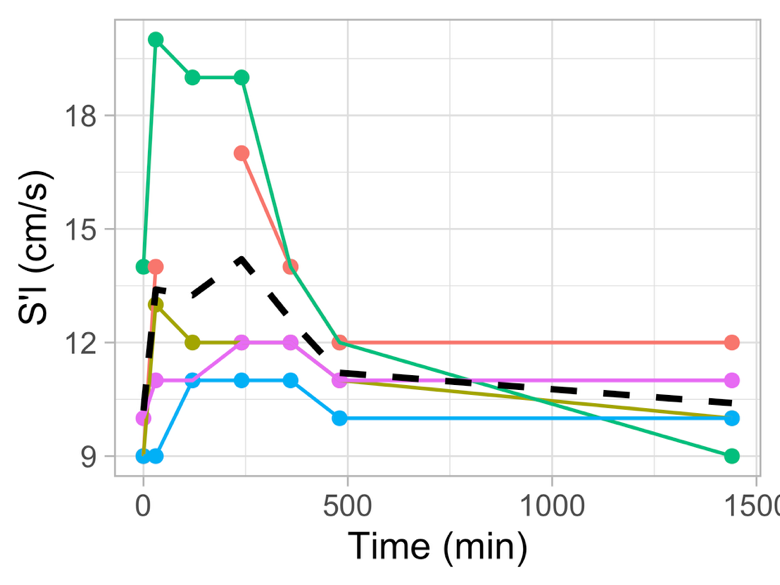
**Supplementary Figure C. Evolution of peak systolic velocity of the lateral mitral annulus over time following intravenous pimobendan administration in five healthy awake Beagle dogs**

Pimobendan (0.15 mg/kg IV) was administered immediately after the baseline (T0) measurements. Each color represents an individual dog. The dashed black line represents the mean. *S’l, peak systolic velocity of the lateral mitral annulus.*

**Supplementary Table B. Peak systolic velocity of the lateral mitral annulus over time: results of linear mixed models.**

| Dependent variable | Independent Variable | Estimate | 95% confidence interval |
| --- | --- | --- | --- |
| S’l (cm/s) | **T30m** | **3.20** | **1.03; 5.37** |
|  | **T2h** | **3.30** | **0.94; 5.58** |
|  | **T4h** | **4.00** | **1.83; 6.17** |
|  | **T6h** | **2.40** | **0.23; 4.57** |
|  | T8h | 1.00 | -1.17; 3.17 |
|  | T24h | 0.20 | -1.97; 2.37 |

Pimobendan (0.15 mg/kg IV) was administered immediately after the baseline (T0) measurements in five healthy awake Beagle dogs. The values at different time points were compared to those at T0 using a linear mixed model, with the dog’s name included as a random effect. In the table, the estimate represents the mean difference between each time point and T0. The estimate is presented along with its 95% confidence interval. Significant differences are indicated in bold. *S’l, peak systolic velocity of the lateral mitral annulus.*


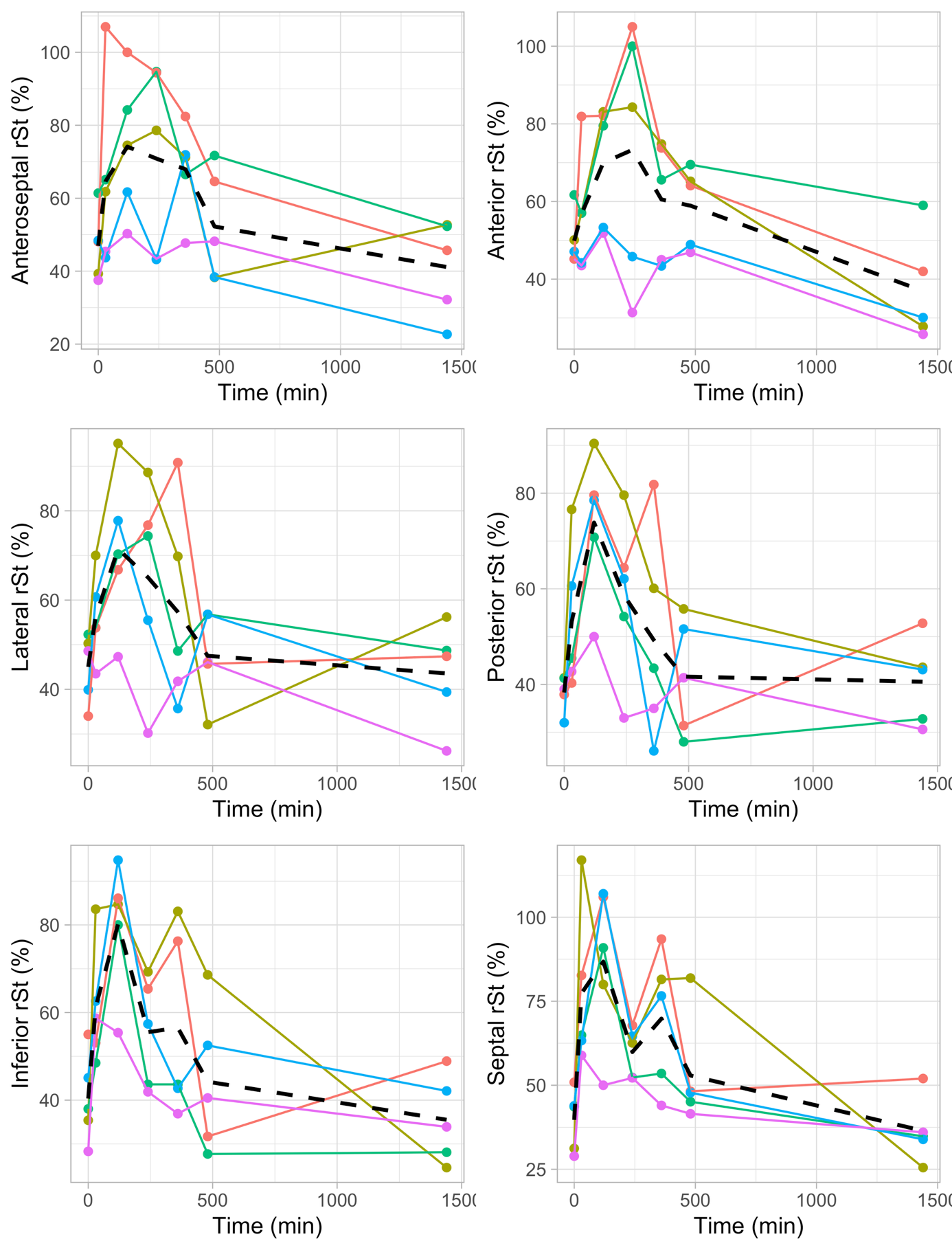
**Supplementary Figure D. Evolution of radial systolic strain over time following intravenous pimobendan administration in five healthy awake Beagle dogs.**

Pimobendan (0.15 mg/kg IV) was administered immediately after the baseline (T0) measurements. Each color represents an individual dog. The dashed black line represents the mean. All strain values are expressed as absolute values. *Anterospetal rSt, rSt at the anteroseptal segment; Anterior rSt, rSt at the anterior segment; Lateral rSt, rSt at the lateral segment; Posterior rSt, rSt at the posterior segment; Inferior rSt, rSt at the interior segment; Septal rSt, rSt at the septal segment.*

**Supplementary Table C. Radial systolic strain parameters of the 6 left ventricular segments at the level of the papillary muscles over time: results of linear mixed models.**

| Dependent variable | Independent Variable | Estimate | 95% confidence interval |
| --- | --- | --- | --- |
| Anteroseptal rSt (%) | **T30m** | **18** | **4; 31** |
|  | **T2h** | **27** | **14; 41** |
|  | **T4h** | **23** | **10; 38** |
|  | **T6h** | **21** | **7; 35** |
|  | T8h | 5 | -8; 19 |
|  | T24h | -6 | -20; 8 |
| Anterior rSt (%) | T30m | 7 | -6; 20 |
|  | **T2h** | **20** | **7; 33** |
|  | **T4h** | **23** | **10; 36** |
|  | T6h | 11 | -2; 24 |
|  | T8h | 9 | -4; 22 |
|  | T24h | -12 | -26; 0 |
| Lateral rSt (%) | T30m | 11 | -4; 27 |
|  | **T2h** | **26** | **11; 42** |
|  | **T4h** | **20** | **5; 36** |
|  | T6h | 12 | -3; 28 |
|  | T8h | 2 | -13; 18 |
|  | T24h | -1 | -17; 14 |
| Posterior rSt (%) | **T30m** | **15** | **1; 28** |
|  | **T2h** | **36** | **22; 49** |
|  | **T4h** | **20** | **7; 34** |
|  | T6h | 11 | -3; 24 |
|  | T8h | 3 | -10; 17 |
|  | T24h | 2 | -11; 16 |
| Inferior rSt (%) | **T30m** | **21** | **7; 35** |
|  | **T2h** | **40** | **26; 53** |
|  | **T4h** | **15** | **2; 29** |
|  | **T6h** | **16** | **3; 30** |
|  | T8h | 4 | -10; 17 |
|  | T24h | -5 | -18; 9 |
| Septal rSt (%) | **T30m** | **38** | **21; 54** |
|  | **T2h** | **47** | **31, 63** |
|  | **T4h** | **20** | **4; 36** |
|  | **T6h** | **30** | **14; 46** |
|  | T8h | 13 | -3; 29 |
|  | T24h | -3 | -20; 13 |

Pimobendan (0.15 mg/kg IV) was administered immediately after the baseline (T0) measurements in five healthy awake Beagle dogs. The values at different time points were compared to those at T0 using a linear mixed model, with the dog’s name included as a random effect. In the table, the estimate represents the mean difference between each time point and T0. The estimate is presented along with its 95% confidence interval. Significant differences are indicated in bold. All strain values are expressed as absolute values. *rSt, radial strain.*


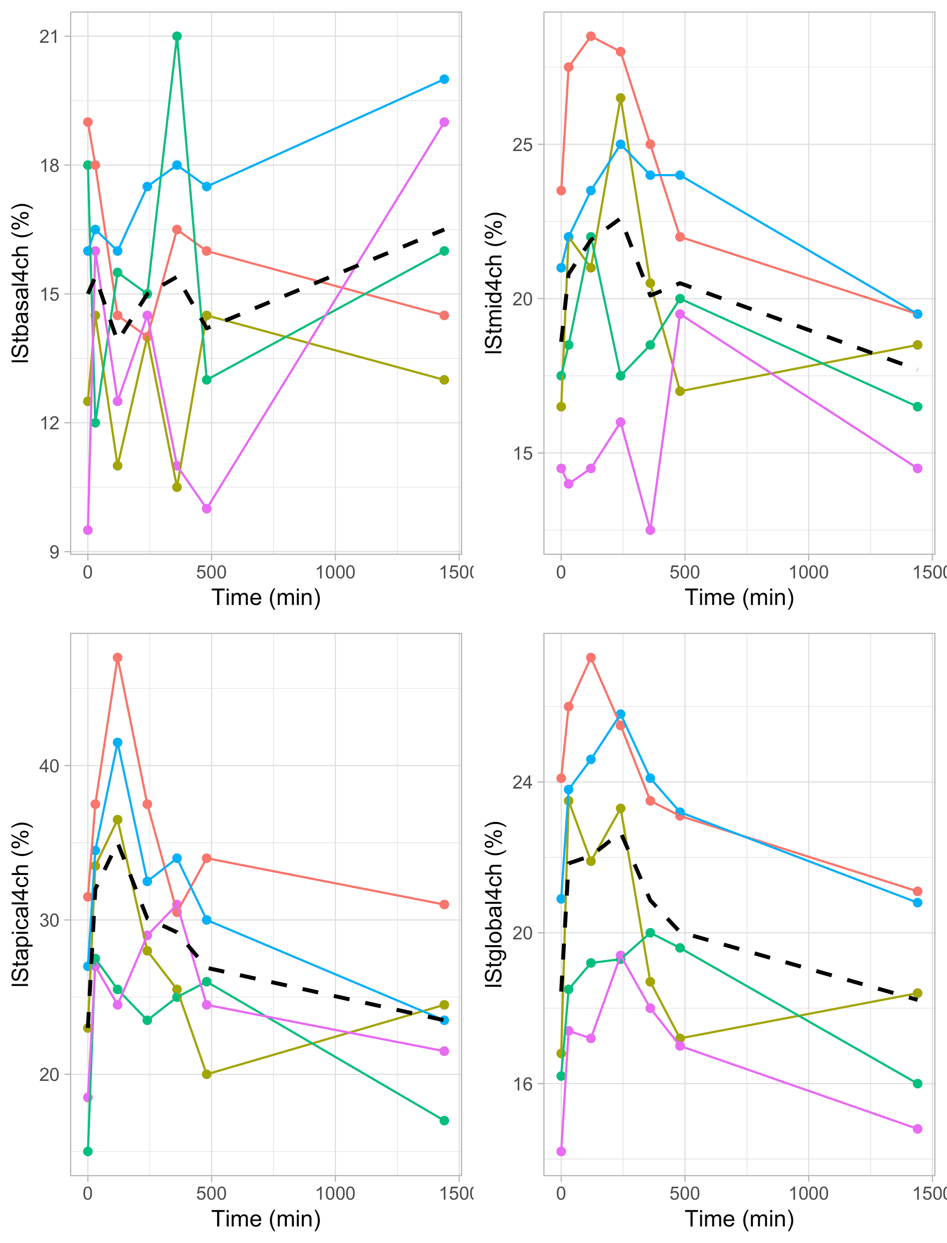
**Supplementary Figure E. Evolution of longitudinal systolic strain obtained from the left apical 4-chamber view over time following intravenous pimobendan administration in five healthy awake Beagle dogs.**

Pimobendan (0.15 mg/kg IV) was administered immediately after the baseline (T0) measurements. Each color represents an individual dog. The dashed black line represents the mean. All strain values are expressed as absolute values. *lStapical4ch, lSt averaged from apical septal and apical lateral segments on the left apical 4-chamber view; lStbasal4ch, lSt averaged from basal septal and basal lateral segments on the left apical 4-chamber view; lStglobal4ch, mean of lStapical4ch, lStbasal4ch and lStmid4ch; lStmid4ch, lSt averaged from mid septal and mid lateral segments on the left apical 4-chamber view.*

**Supplementary Table D. Evolution of longitudinal systolic strain parameters over time derived from the left apical four-chamber view according to left ventricular level: results of linear mixed models.**

| Dependent variable | Independent Variable | Estimate | 95% confidence interval |
| --- | --- | --- | --- |
| lStbasal4ch (%) | T30m | 0 | -3; 3 |
|  | T2h | -1 | -4; 2 |
|  | T4h | 0 | -3; 3 |
|  | T6h | 0 | -3; 3 |
|  | T8h | -1 | -4; 2 |
|  | T24h | 1.5 | -1; 4 |
| lStmid4ch (%) | T30m | 2 | 0.7; 5 |
|  | **T2h** | **3** | **1; 6** |
|  | **T4h** | **4** | **1; 7** |
|  | T6h | 2 | -1; 4 |
|  | T8h | 2 | -1; 4 |
|  | T24h | -1 | -3; 2 |
| lStapical4ch (%) | **T30m** | **9** | **5; 13** |
|  | **T2h** | **12** | **8; 16** |
|  | **T4h** | **7** | **3; 11** |
|  | **T6h** | **6** | **2; 10** |
|  | T8h | 4 | -0.05; 8 |
|  | T24h | 1 | -3; 4 |
| lStglobal4ch (%) | T30m | 3 | 2; 5 |
|  | T2h | 4 | 2; 5 |
|  | T4h | 4 | 3; 6 |
|  | T6h | 2 | 1; 4 |
|  | **T8h** | **2** | **0.1; 3** |
|  | T24h | 0 | -2; 1 |

Pimobendan (0.15 mg/kg IV) was administered immediately after the baseline (T0) measurements in five healthy awake Beagle dogs. The values at different time points were compared to those at T0 using a linear mixed model, with the dog’s name included as a random effect. In the table, the estimate represents the mean difference between each time point and T0. The estimate is presented along with its 95% confidence interval. Significant differences are indicated in bold. All strain values are expressed as absolute values. *lStapical4ch, lSt averaged from apical septal and apical lateral segments on the apical 4-chamber view; lStbasal4ch, lSt averaged from basal septal and basal lateral segments on the apical 4-chamber view; lStglobal4ch, mean of lStapical4ch, lStbasal4ch and lStmid4ch; lStmid4ch, lSt averaged from mid septal and mid lateral segments on the apical 4-chamber view.*

**Supplementary Figure F. Evolution of longitudinal systolic strain obtained from the left apical 5-chamber view over time following intravenous pimobendan administration in five healthy awake Beagle dogs.**


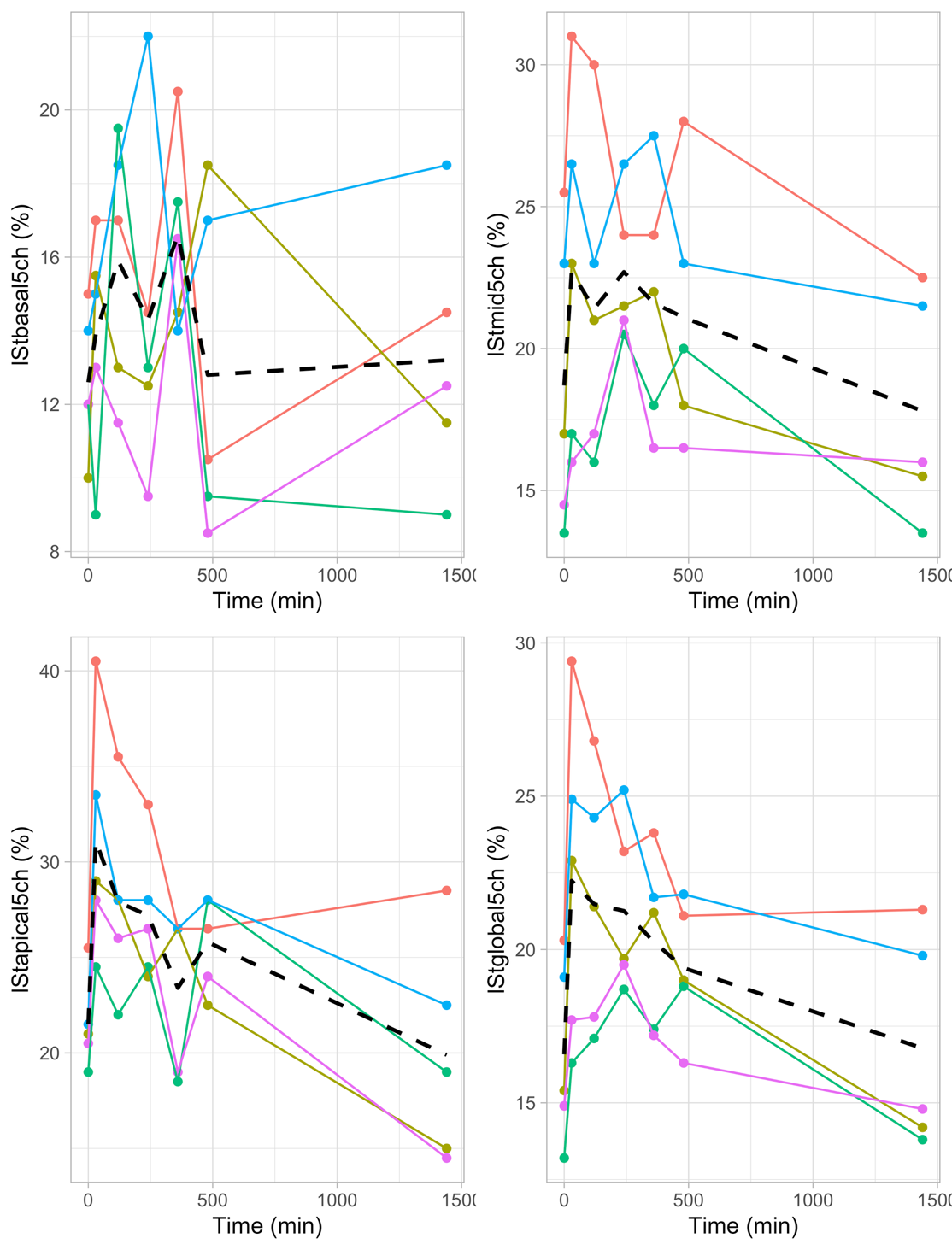


Pimobendan (0.15 mg/kg IV) was administered immediately after the baseline (T0) measurements. Each color represents an individual dog. The dashed black line represents the mean. All strain values are expressed as absolute values. *lStapical5ch, lSt averaged from apical septal and apical lateral segments on the apical 5-chamber view; lStbasal5ch, lSt averaged from basal septal and basal lateral segments on the apical 5-chamber view; lStglobal5ch, mean of lStapical5ch, lStbasal5ch and lStmid5ch; lStmid5ch, lSt averaged from mid septal and mid lateral segments on the apical 5-chamber view.*

**Supplementary Table E. Evolution of longitudinal systolic strain parameters over time derived from the left apical five-chamber view according to left ventricular level: results of linear mixed models.**

| Dependent variable | Independent Variable | Estimate | 95% confidence interval |
| --- | --- | --- | --- |
| lStbasal5ch (%) | T30m | 1 | -2; 5 |
|  | T2h | 3 | -0.3; 7 |
|  | T4h | 2 | -2; 5 |
|  | **T6h** | **4** | **0.4; 8** |
|  | T8h | 0 | -3; 4 |
|  | T24h | 1 | -3; 4 |
| lStmid5ch (%) | **T30m** | **4** | **2; 6** |
|  | **T2h** | **3** | **0.3; 5** |
|  | **T4h** | **4** | **2; 6** |
|  | **T6h** | **3** | **0.5; 5** |
|  | T8h | 2 | -0.1; 5 |
|  | T24h | -1 | -7; 2 |
| lStapical5ch (%) | **T30m** | **10** | **6; 13** |
|  | **T2h** | **6** | **3; 10** |
|  | **T4h** | **6** | **2; 9** |
|  | T6h | 2 | -1; 5 |
|  | T8h | **4** | **1; 8** |
|  | T24h | -2 | -5; 2 |
| lStglobal5ch (%) | **T30m** | **6** | **2; 5** |
|  | **T2h** | **5** | **2; 5** |
|  | **T4h** | **5** | **3; 6** |
|  | **T6h** | **4** | **2; 6** |
|  | **T8h** | **3** | **1; 5** |
|  | T24h | 0 | -2; 2 |

Pimobendan (0.15 mg/kg IV) was administered immediately after the baseline (T0) measurements in five healthy awake Beagle dogs. The values at different time points were compared to those at T0 using a linear mixed model, with the dog’s name included as a random effect. In the table, the estimate represents the mean difference between each time point and T0. The estimate is presented along with its 95% confidence interval. Significant differences are indicated in bold. All strain values are expressed as absolute values. *lStapical5ch, lSt averaged from apical septal and apical lateral segments on the apical 5-chamber view; lStbasal5ch, lSt averaged from basal septal and basal lateral segments on the apical 5-chamber view; lStglobal5ch, mean of lStapical5ch, lStbasal5ch and lStmid5ch; lStmid5ch, lSt averaged from mid septal and mid lateral segments on the apical 5-chamber view.*

**Supplementary Figure G. Evolution of longitudinal systolic strain obtained from the left apical two-chamber view over time following intravenous pimobendan administration in five healthy awake Beagle dogs.**


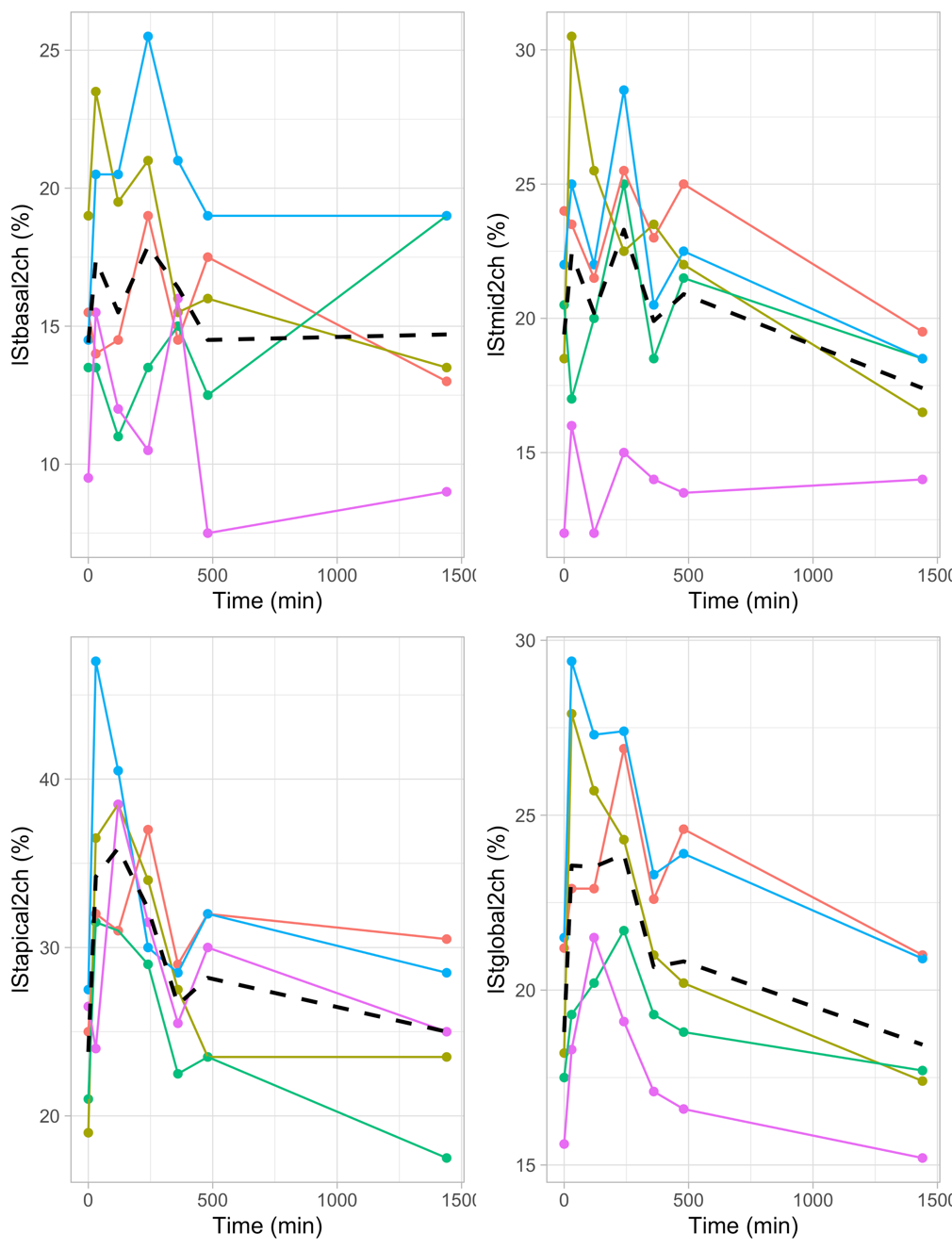


Pimobendan (0.15 mg/kg IV) was administered immediately after the baseline (T0) measurements. Each color represents an individual dog. The dashed black line represents the mean. All strain values are expressed as absolute values. *lStapical2ch, lSt averaged from apical septal and apical lateral segments on the apical 2-chamber view; lStbasal2ch, lSt averaged from basal septal and basal lateral segments on the apical 2-chamber view; lStglobal2ch, mean of lStapical2ch, lStbasal2ch and lStmid2ch; lStmid2ch, lSt averaged from mid septal and mid lateral segments on the apical 2-chamber view.*

**Supplementary Table F. Evolution of longitudinal systolic strain parameters over time derived from the left apical two-chamber view according to left ventricular level: results of linear mixed models.**

| Dependent variable | Independent Variable | Estimate | 95% confidence interval |
| --- | --- | --- | --- |
| lStbasal2ch (%) | T30m | 3 | -0.3; 6 |
|  | T2h | 1 | -2; 4 |
|  | **T4h** | **4** | **0.2; 7** |
|  | T6h | 2 | -1; 5 |
|  | T8h | 0 | -3; 3 |
|  | T24h | 0 | -3; 4 |
| lStmid2ch (%) | T30m | 3 | 0.1; 6 |
|  | T2h | 1 | -2; 4 |
|  | **T4h** | **4** | **1;7** |
|  | T6h | 1 | -2; 3 |
|  | T8h | 2 | -1; 4 |
|  | T24h | -2 | -5; 1 |
| lStapical2ch (%) | **T30m** | **10** | **6; 15** |
|  | **T2h** | **12** | **7; 17** |
|  | **T4h** | **9** | **4; 13** |
|  | T6h | 3 | -2; 8 |
|  | T8h | 4 | -0.3; 9 |
|  | T24h | 1 | -4; 6 |
| lStglobal2ch (%) | **T30m** | **5** | **2; 5** |
|  | **T2h** | **5** | **2; 5** |
|  | **T4h** | **5** | **3; 6** |
|  | **T6h** | **2** | **1; 4** |
|  | **T8h** | **2** | **0.1; 3** |
|  | T24h | 0 | -2; 1 |

Pimobendan (0.15 mg/kg IV) was administered immediately after the baseline (T0) measurements in five healthy awake Beagle dogs. The values at different time points were compared to those at T0 using a linear mixed model, with the dog’s name included as a random effect. In the table, the estimate represents the mean difference between each time point and T0. The estimate is presented along with its 95% confidence interval. Significant differences are indicated in bold. All strain values are expressed as absolute values. *lStapical2ch, lSt averaged from apical septal and apical lateral segments on the apical 2-chamber view; lStbasal2ch, lSt averaged from basal septal and basal lateral segments on the apical 2-chamber view; lStglobal2ch, mean of lStapical2ch, lStbasal2ch and lStmid2ch; lStmid2ch, lSt averaged from mid septal and mid lateral segments on the apical 2-chamber view.*

**Supplementary Figure H. Distribution of segmental longitudinal strain (basal, mid, apical) across all time points.**


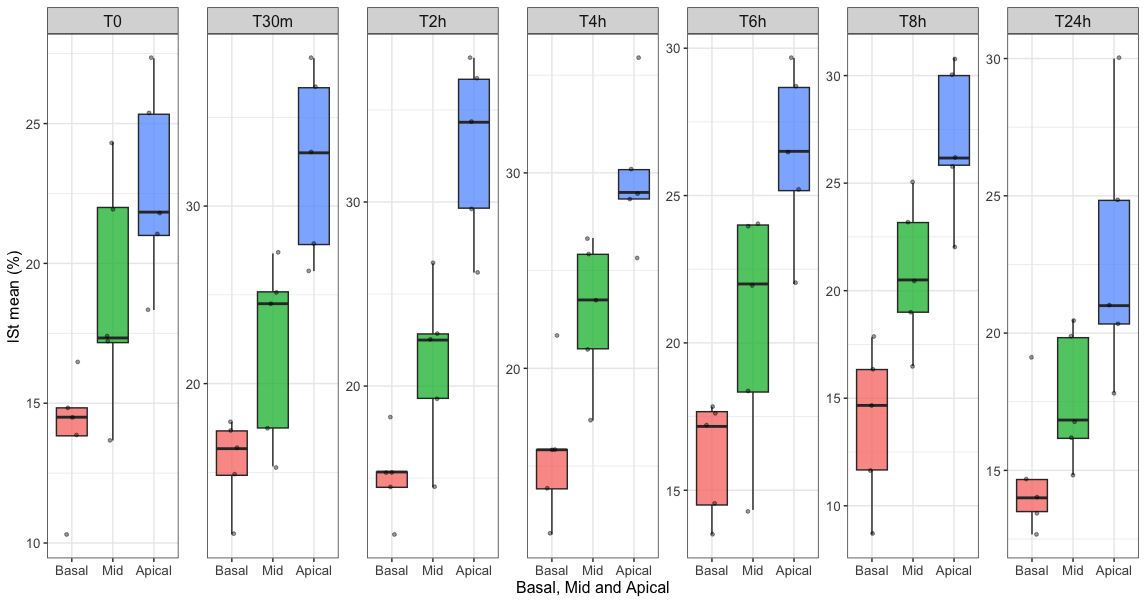


Pimobendan (0.15 mg/kg IV) was administered immediately after the baseline (T0) measurements in five healthy awake Beagle dogs. Basal corresponds to lStbasalmean, Mid corresponds to lStmidmean, and Apical corresponds to lStapicalmean. All strain values are expressed as absolute values. *lStbasalmean, mean of lStbasal from the apical 2-, 4-, and 5-chamber views; lStmidmean, mean of lStmid from the apical 2-, 4-, and 5-chamber views; lStapicalmean, mean of lStapical from the apical 2-, 4-, and 5-chamber views.*

**
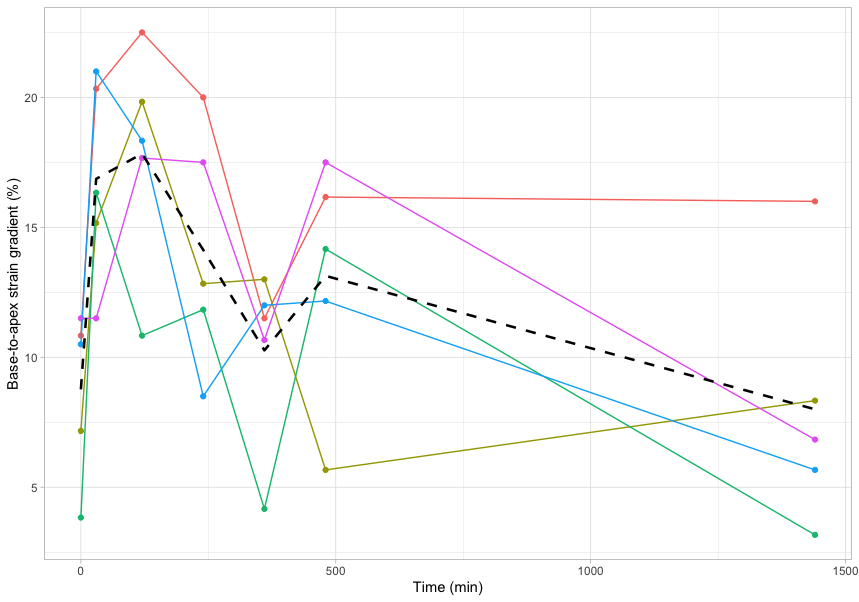
Supplementary Figure I. Evolution of the base-to-apex systolic strain gradient following intravenous pimobendan administration in five healthy awake Beagle dogs.**

Pimobendan (0.15 mg/kg IV) was administered immediately after the baseline (T0). The gradient was calculated for each dog as lStapicalmean − lStbasalmean, where lStbasalmean represents the mean of lStbasal from the apical 2-, 4-, and 5-chamber views, and lStapicalmean the mean of lStapical from the apical 2-, 4-, and 5-chamber views. Each color represents an individual dog. The dashed black line represents the mean. All strain values are expressed as absolute values.

**Supplementary Table G. Evolution of the base-to-apex systolic strain gradient over time: results of linear mixed models.**

| Dependent variable | Independent Variable | Estimate | 95% confidence interval |
| --- | --- | --- | --- |
| Base-to-apex strain gradient (%) | **T30m** | **8** | **4; 12** |
|  | **T2h** | **9** | **5; 13** |
|  | **T4h** | **5** | **1; 9** |
|  | T6h | 2 | -2; 5 |
|  | T8h | 4 | 0; 8 |
|  | T24h | -1 | -5; 3 |

Pimobendan (0.15 mg/kg IV) was administered immediately after the baseline (T0) measurements in five healthy awake Beagle dogs. The gradient was calculated for each dog as lStapicalmean − lStbasalmean, where lStbasalmean represents the mean of lStbasal from the apical 2-, 4-, and 5-chamber views, and lStapicalmean the mean of lStapical from the apical 2-, 4-, and 5-chamber views. All strain values are expressed as absolute values.

**Supplementary Table H. Evolution of left atrial parameters over time: results of linear mixed models.**

| Dependent variable | Independent Variable | Estimate | 95% confidence interval |
| --- | --- | --- | --- |
| LAFS (%) | **T30m** | **17** | **12; 23** |
|  | **T2h** | **16** | **10; 21** |
|  | **T4h** | **11** | **6; 17** |
|  | **T6h** | **8** | **3; 14** |
|  | T8h | 5 | -0.1; 11 |
|  | T24h | 2 | -3; 9 |
| LADVol (mL) | **T30m** | **-2.33** | **-3.09; -1.57** |
|  | **T2h** | **-1.89** | **-2.66; -1.13** |
|  | **T4h** | **-1.11** | **-1.87; -0.35** |
|  | T6h | -0.22 | -0.99; 0.54 |
|  | T8h | -0.28 | -1.05; 0.48 |
|  | T24h | -0.23 | -0.99; 0.53 |
| LASVol (mL) | **T30m** | **-1.08** | **-2.04; -0.12** |
|  | T2h | -0.58 | -1.54; 0.38 |
|  | **T4h** | **-1.16** | **-2.12; -0.20** |
|  | T6h | -0.52 | -1.48; 0.45 |
|  | T8h | -0.22 | -1.18; 0.74 |
|  | T24h | 0.34 | -0.62; 1.30 |
| LAEF (%) | **T30m** | **26** | **17; 35** |
|  | **T2h** | **21** | **12; 30** |
|  | T4h | 7 | -1; 16 |
|  | T6h | -2 | -11; 7 |
|  | T8h | 2 | -6; 11 |
|  | T24h | 4 | -4; 13 |
| LAS:Ao | T30m | -0.01 | -0.09; 0.07 |
|  | T2h | -0.01 | -0.09; 0.07 |
|  | T4h | 0.04 | -0.04; 0.12 |
|  | T6h | -0.01 | -0.09; 0.07 |
|  | T8h | 0.00 | -0.08; 0.08 |
|  | T24h | 0.01 | -0.07; 0.09 |
| LAD:Ao | T30m | -0.05 | -0.13; 0.02 |
|  | T2h | -0.06 | -0.13; 0.02 |
|  | T4h | -0.02 | -0.10; 0.05 |
|  | T6h | 0.01 | -0.06; 0.09 |
|  | T8h | 0.00 | -0.07; 0.08 |
|  | T24h | -0.01 | -0.08; 0.07 |

Pimobendan (0.15 mg/kg IV) was administered immediately after the baseline (T0) measurements in five healthy awake Beagle dogs. The values at different time points were compared to those at T0 using a linear mixed model, with the dog’s name included as a random effect. In the table, the estimate represents the mean difference between each time point and T0. The estimate is presented along with its 95% confidence interval. Significant differences are indicated in bold. *LAD:Ao, end-diastolic left-atrium-to-aorta ratio; LAS:Ao, end-systolic left-atrium-to-aorta ratio; LADVol, end-diastolic left atrial volume; LAEF, left atrial ejection fraction; LAFS, left atrial fractional shortening; LASVol, end-systolic left atrial volume.*

**Supplementary Figure J. Evolution of left atrial parameters over time following intravenous pimobendan administration in five healthy awake Beagle dogs.**


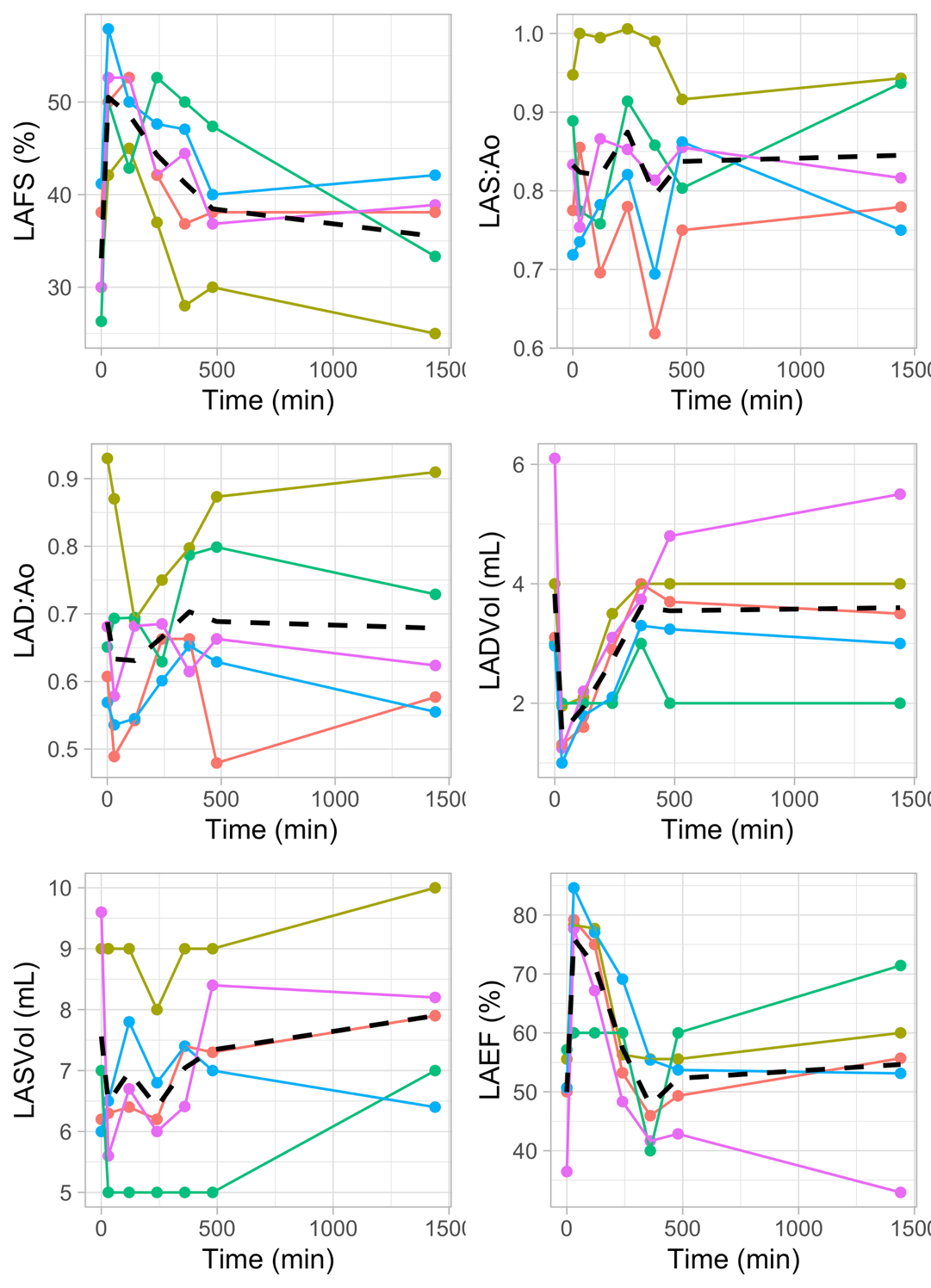


Pimobendan (0.15 mg/kg IV) was administered immediately after the baseline (T0) measurements. Each color represents an individual dog. The dashed black line represents the mean. *LAD:Ao, end-diastolic left-atrium-to-aorta ratio; LAS:Ao, end-systolic left-atrium-to-aorta ratio; LADVol, end-diastolic left atrial volume; LAEF, left atrial ejection fraction; LAFS, left atrial fractional shortening; LASVol, end-systolic left atrial volume.*

**Supplementary Figure K. Evolution of left heart diastolic function parameters over time following intravenous pimobendan administration in five healthy awake Beagle dogs.**


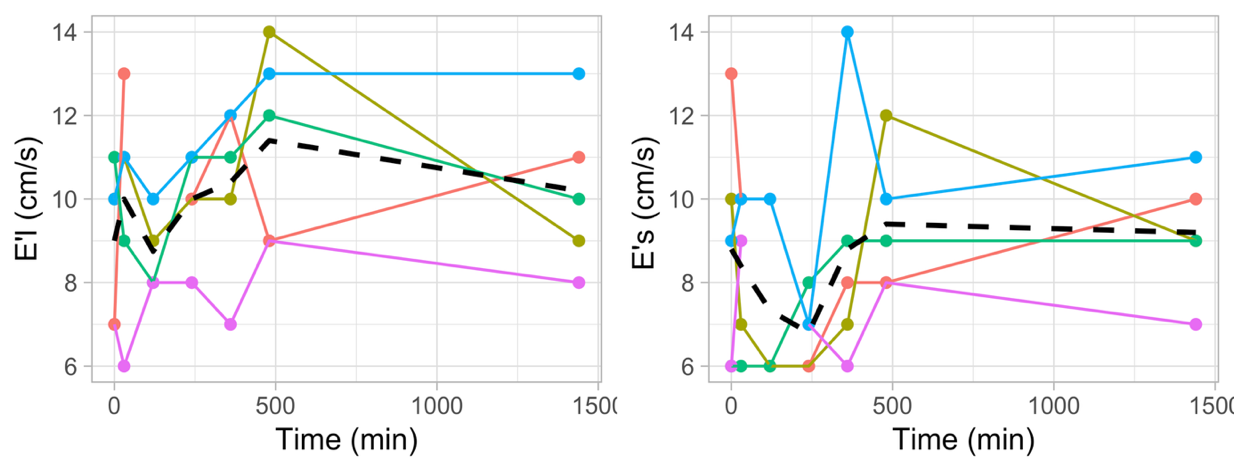

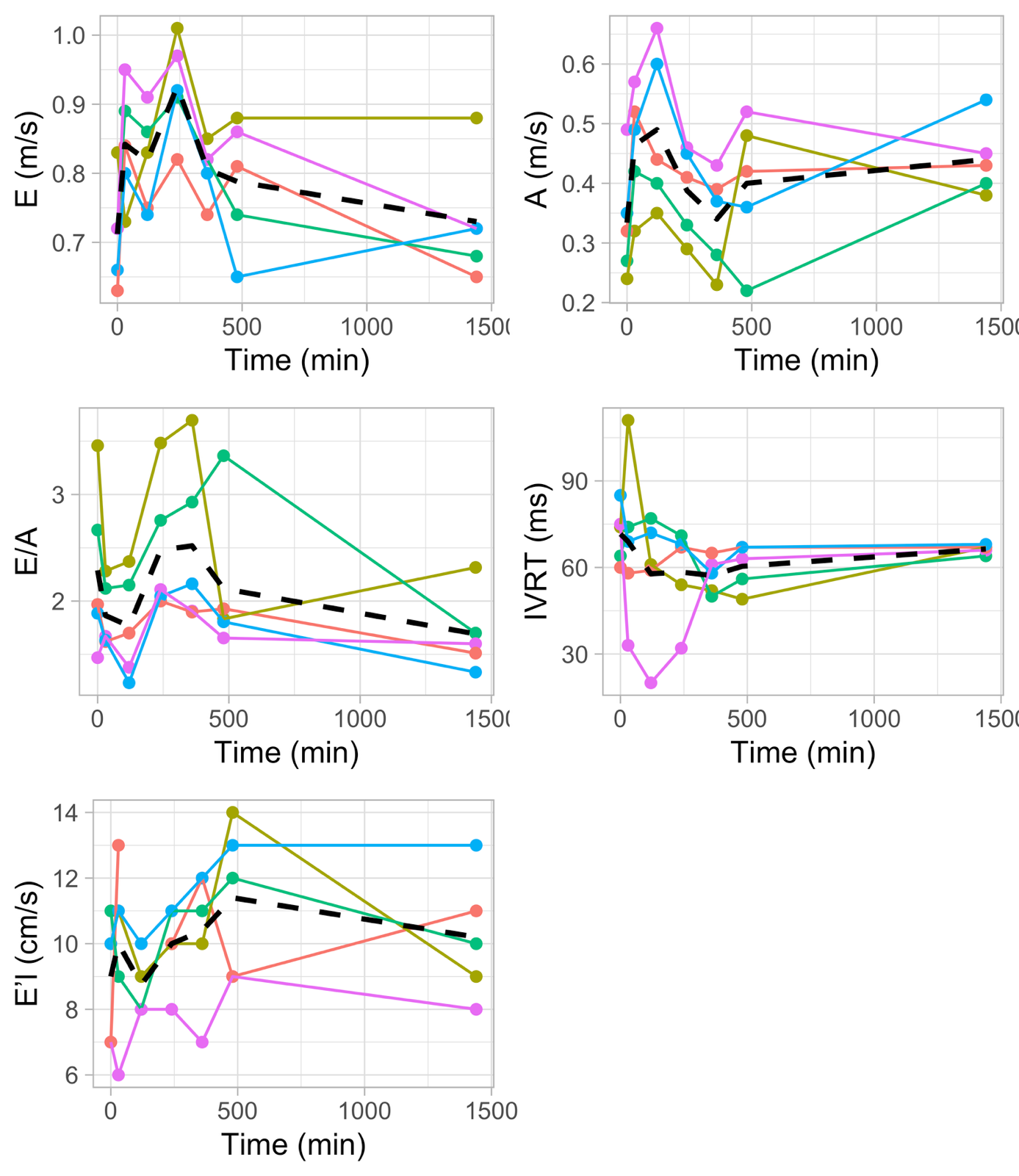


Pimobendan (0.15 mg/kg IV) was administered immediately after the baseline (T0) measurements. Each color represents an individual dog. The dashed black line represents the mean. *A, late diastolic mitral flow velocity; E, early diastolic mitral flow velocity; E/A, early-to-late diastolic mitral flow velocity ratio; E’s, peak early diastolic velocity of the septal mitral annulus; E’l, peak early diastolic velocity of the lateral mitral annulus; IVRT, isovolumetric relaxation time*

**
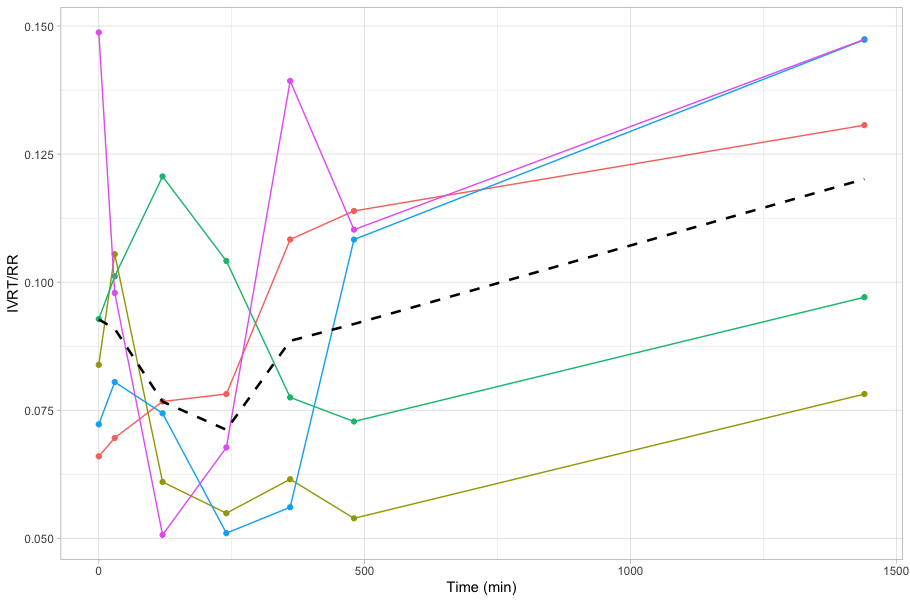
Supplementary Figure L Evolution of heart rate-corrected isovolumetric relaxation time over time following intravenous pimobendan administration in five healthy awake Beagle dogs.**

Pimobendan (0.15 mg/kg IV) was administered immediately after the baseline (T0) measurements. Each color represents an individual dog. The dashed black line represents the mean. IVRT/RR, isovolumetric relaxation time normalized to the RR interval, calculated as IVRT (ms) divided by RR interval (ms).

**Supplementary Table I. Evolution of left heart diastolic function parameters over time: results of linear mixed models.**

| Dependent variable | Independent Variable | Estimate | 95% confidence interval |
| --- | --- | --- | --- |
| E (m/s) | **T30m** | **0.13** | **0.06; 0.19** |
|  | **T2h** | **0.11** | **0.03; 0.18** |
|  | **T4h** | **0.21** | **0.14; 0.29** |
|  | **T6h** | **0.09** | **0.02; 0.17** |
|  | **T8h** | **0.08** | **0.004; 0.15** |
|  | T24h | 0.02 | -0.05; 0.09 |
| A (m/s) | **T30m** | **0.13** | **0.06; 0.20** |
|  | **T2h** | **0.16** | **0.09; 0.23** |
|  | T4h | 0.05 | -0.02; 0.12 |
|  | T6h | 0.01 | -0.06; 0.08 |
|  | T8h | 0.06 | -0.005; 0.14 |
|  | **T24h** | **0.11** | **0.02; 0.19** |
| E/A | **T30m** | **-0.43** | **-0.84; -0.01** |
|  | **T2h** | **-0.52** | **-0.94; -0.11** |
|  | T4h | 0.19 | -0.23; 0.60 |
|  | T6h | 0.23 | -0.19; 0.64 |
|  | T8h | -0.17 | -0.59; 0.24 |
|  | **T24h** | **-0.60** | **-1.01; -0.18** |
| IVRT (ms) | T30m | -2.60 | -19.83; 14.63 |
|  | T2h | -13.80 | -31.03; 3.43 |
|  | T4h | -13.20 | -30.43; 4.03 |
|  | T6h | -14.40 | -31.63; 2.83 |
|  | T8h | -11.20 | -28.43; 6.03 |
|  | T24h | -5.20 | -22.43; 12.03 |
| E’l (cm/s) | T30m | -0.40 | -2.56; 1.76 |
|  | T2h | -1.63 | -4.15; 0.91 |
|  | T4h | -2.00 | -4.16; 0.16 |
|  | T6h | 0.00 | -2.16; 2.16 |
|  | T8h | 0.60 | -1.56; 2.76 |
|  | T24h | 0.40 | -1.76; 2.56 |
| E’s (cm/s) | T30m | -0.40 | -2.56; 1.76 |
|  | T2h | -1.63 | -4.15; 0.91 |
|  | T4h | -2.00 | -4.16; 0.16 |
|  | T6h | 0.00 | -2.16; 2.16 |
|  | T8h | 0.60 | -1.56; 2.76 |
|  | T24h | 0.40 | -1.76; 2.56 |

Pimobendan (0.15 mg/kg IV) was administered immediately after the baseline (T0) measurements in five healthy awake Beagle dogs. The values at different time points were compared to those at T0 using a linear mixed model, with the dog’s name included as a random effect. In the table, the estimate represents the mean difference between each time point and T0. The estimate is presented along with its 95% confidence interval. Significant differences are indicated in bold. *A, late diastolic mitral flow velocity; E, early diastolic mitral flow velocity; E/A, early-to-late diastolic mitral flow velocity ratio; E’l, peak early diastolic velocity of the lateral mitral annulus; E’s, peak early diastolic velocity of the septal mitral annulus; IVRT, isovolumetric relaxation time; LAD:Ao, end-diastolic left-atrium-to-aorta ratio.*

**Supplementary Table J. Evolution of heart rate-corrected isovolumetric relaxation time over time: results of linear mixed models.**

| Dependent variable | Independent Variable | Estimate | 95% confidence interval |
| --- | --- | --- | --- |
| IVRT/RR | T30m | 0.00 | -0.03; 0.03 |
|  | T2h | -0.02 | -0.05; 0.02 |
|  | T4h | -0.02 | -0.05; 0.01 |
|  | T6h | -0.01 | -0.03; 0.03 |
|  | T8h | 0.00 | -0.03; 0.03 |
|  | T24h | 0.03 | -0.002; 0.06 |

Pimobendan (0.15 mg/kg IV) was administered immediately after the baseline (T0) measurements in five healthy awake Beagle dogs. The values at different time points were compared to those at T0 using a linear mixed model, with the dog’s name included as a random effect. In the table, the estimate represents the mean difference between each time point and T0. The estimate is presented along with its 95% confidence interval. IVRT/RR, isovolumetric relaxation time normalized to the RR interval, calculated as IVRT (ms) divided by RR interval (ms).


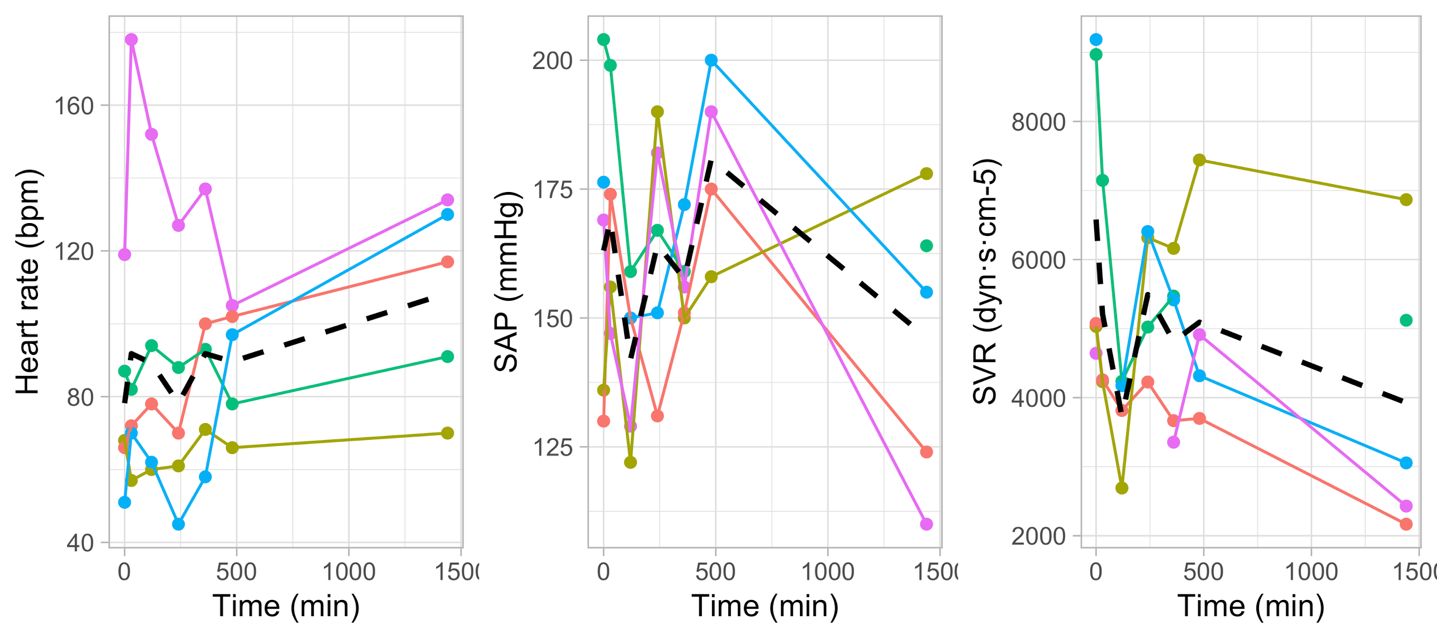
**Supplementary Figure M. Evolution of heart rate, systolic arterial blood pressure and systemic vascular resistance over time following intravenous pimobendan administration in five healthy awake Beagle dogs.**

Pimobendan (0.15 mg/kg IV) was administered immediately after the baseline (T0) measurements. Each color represents an individual dog. The dashed black line represents the mean. *SAP: systolic arterial pressure, SVR: systemic vascular resistance.*

**Supplementary Table K. Evolution of heart rate, blood pressure and systemic vascular resistance over time: results of linear mixed models.**

| Dependent variable | Independent Variable | Estimate | 95% confidence interval |
| --- | --- | --- | --- |
| Heart rate (bpm) | T30m | 14 | -8; 35 |
|  | T2h | 11 | -10; 32 |
|  | T4h | 50 | -21; 21 |
|  | T6h | 14 | -8; 35 |
|  | T8h | 11 | -10; 33 |
|  | **T24h** | **30** | **9; 52** |
| Systolic arterial pressure (mmHg) | T30m | 7 | -18; 32 |
|  | T2h | -21 | -44; 2 |
|  | T4h | 1 | -29; 18 |
|  | T6h | -5 | -5; 45 |
|  | T8h | 20 | -5; 45 |
|  | T24h | -17 | -40; 6 |
| Systemic vascular resistance (dyn·s·cm⁻⁵) | T30m | -1527 | -3320; 289 |
|  | **T2h** | **-3067** | **-4701; -1405** |
|  | T4h | -1302 | -2937; 358 |
|  | **T6h** | **-1767** | **-3300; -233** |
|  | T8h | -1279 | -2934; 351 |
|  | **T24h** | **-2653** | **-4186; -1119** |

Pimobendan (0.15 mg/kg IV) was administered immediately after the baseline (T0) measurements in five healthy awake Beagle dogs. The values at different time points were compared to those at T0 using a linear mixed model, with the dog’s name included as a random effect. In the table, the estimate represents the mean difference between each time point and T0. The estimate is presented along with its 95% confidence interval. Significant differences are indicated in bold.

**Reference**

1. Chetboul V, Athanassiadis N, Concordet D, Nicolle A, Tessier D, Castagnet M, et al. Observer-dependent variability of quantitative clinical endpoints: the example of canine echocardiography. J Vet Pharmacol Ther. 2004 Feb;27(1):49–56. doi:10.1046/j.0140-7783.2003.00543.x PubMed PMID: 14995967.

2. Misbach C, Lefebvre HP, Concordet D, Gouni V, Trehiou-Sechi E, Petit AMP, et al. Echocardiography and conventional Doppler examination in clinically healthy adult Cavalier King Charles Spaniels: effect of body weight, age, and gender, and establishment of reference intervals. J Vet Cardiol Off J Eur Soc Vet Cardiol. 2014 Jun;16(2):91–100. doi:10.1016/j.jvc.2014.03.001 PubMed PMID: 24834861.

3. Hansson K, Häggström J, Kvart C, Lord P. Left atrial to aortic root indices using two-dimensional and M-mode echocardiography in cavalier King Charles spaniels with and without left atrial enlargement. Vet Radiol Ultrasound Off J Am Coll Vet Radiol Int Vet Radiol Assoc. 2002;43(6):568–75. doi:10.1111/j.1740-8261.2002.tb01051.x PubMed PMID: 12502113.

4. Dickson D, Caivano D, Matos JN, Summerfield N, Rishniw M. Two-dimensional echocardiographic estimates of left atrial function in healthy dogs and dogs with myxomatous mitral valve disease. J Vet Cardiol Off J Eur Soc Vet Cardiol. 2017 Dec;19(6):469–79. doi:10.1016/j.jvc.2017.09.003 PubMed PMID: 29111284.

5. Sahn DJ, DeMaria A, Kisslo J, Weyman A. Recommendations regarding quantitation in M-mode echocardiography: results of a survey of echocardiographic measurements. Circulation. 1978 Dec;58(6):1072–83. doi:10.1161/01.cir.58.6.1072 PubMed PMID: 709763.

6. Thomas WP, Gaber CE, Jacobs GJ, Kaplan PM, Lombard CW, Moise NS, et al. Recommendations for standards in transthoracic two-dimensional echocardiography in the dog and cat. Echocardiography Committee of the Specialty of Cardiology, American College of Veterinary Internal Medicine. J Vet Intern Med. 1993;7(4):247–52. doi:10.1111/j.1939-1676.1993.tb01015.x PubMed PMID: 8246215.

7. Bonagura JD, Visser LC. Echocardiographic assessment of dilated cardiomyopathy in dogs. J Vet Cardiol Off J Eur Soc Vet Cardiol. 2022 Apr;40:15–50. doi:10.1016/j.jvc.2021.08.004 PubMed PMID: 34750089.

8. Chetboul V, Poissonnier C, Foulex P, Alvarado MP, Trehiou-Sechi É, Saponaro V, et al. Volumetric quantification identifies some left atrial dilations undetected by left atrium:aorta ratio measurements: A prospective echocardiographic study in 155 Cavalier King Charles Spaniels with and without degenerative mitral valve disease. PloS One. 2024;19(3):e0300827. doi:10.1371/journal.pone.0300827 PubMed PMID: 38547236; PubMed Central PMCID: PMC10977888.

9. Höllmer M, Willesen JL, Tolver A, Koch J. Left atrial volume and phasic function in clinically healthy dogs of 12 different breeds. Vet J Lond Engl 1997. 2013 Sep;197(3):639–45. doi:10.1016/j.tvjl.2013.05.045 PubMed PMID: 23838204.

10. Serres F, Chetboul V, Tissier R, Gouni V, Desmyter A, Sampedrano CC, et al. Quantification of pulmonary to systemic flow ratio by a Doppler echocardiographic method in the normal dog: Repeatability, reproducibility, and reference ranges. J Vet Cardiol Off J Eur Soc Vet Cardiol. 2009 Jun;11(1):23–9. doi:10.1016/j.jvc.2009.04.001 PubMed PMID: 19467624.

11. Chetboul V, Taton C. Autres techniques d’imagerie ultrasonore cardiovasculaire. In: Encyclopédie d’imagerie cardiovasculaire ultrasonore du chien et du chat. Elsevier Masson. Issy-les-Moulineaux; 2018. p. 145–207.

12. Chetboul V, Serres F, Gouni V, Tissier R, Pouchelon JL. Radial strain and strain rate by two-dimensional speckle tracking echocardiography and the tissue velocity based technique in the dog. J Vet Cardiol. 2007 Nov 1;9(2):69–81. doi:10.1016/j.jvc.2006.11.002

13. Chetboul V, Gouni V, Sampedrano CC, Tissier R, Serres F, Pouchelon JL. Assessment of regional systolic and diastolic myocardial function using tissue Doppler and strain imaging in dogs with dilated cardiomyopathy. J Vet Intern Med. 2007;21(4):719–30. doi:10.1892/0891-6640(2007)21[719:aorsad]2.0.co;2 PubMed PMID: 17708391.

14. Hertzsch S, Wess G. Two-dimensional speckle tracking-derived global longitudinal strain in healthy Doberman Pinschers: method evaluation, variability, and reference values. J Vet Cardiol Off J Eur Soc Vet Cardiol. 2023 Feb;45:3–14. doi:10.1016/j.jvc.2022.11.002 PubMed PMID: 36587449.
