# Supplementary material for: Characterization of global and regional left heart function using advanced echocardiography following intravenous administration of pimobendan in awake dogs
Source: Front Vet Sci. 2026 Jul 3;13:1842861. doi: 10.3389/fvets.2026.1842861 (PMC13375486; doi:10.3389/fvets.2026.1842861)
Supplement: Supplementary file 2 [file Data_Sheet_2.docx]

**Sensitivity analysis**

**Temporal modelling of myocardial deformation parameters using natural cubic splines in a mixed-effects framework**

**Aims** 

The primary analyses reported in the main manuscript modelled time as a categorical variable, allowing pairwise comparisons of each strain parameter against baseline at each individual time point. This approach, while appropriate for detecting discrete changes from baseline, does not formally characterise the continuous temporal trajectory of the pharmacodynamic response. The present sensitivity analysis was therefore conducted to assess the robustness of the main findings by modelling time as a continuous variable using natural cubic splines within the same linear mixed-effects framework, and to evaluate whether the temporal patterns identified in the primary analysis were consistent with those produced by a continuous modelling approach. Given the large number of echocardiographic variables examined in the main manuscript, this sensitivity analysis was restricted to strain parameters, which were considered of particular pharmacodynamic interest as the primary determinants of stroke volume identified in the main analysis.

**Methods**

All strain parameters analysed in the primary manuscript were re-examined using linear mixed-effects models in which the effect of time was represented by a natural cubic spline with three degrees of freedom, implemented using the *ns()* function from the *splines* package, within the *lme4* modelling framework. Three degrees of freedom were selected as a compromise between model flexibility and parsimony, given the limited number of measurement time points (n = 7: T0, T30min, T2h, T4h, T6h, T8h, T24h) and the small sample size (n = 5 dogs). The random-effects structure was identical to that of the primary models, with a random intercept per animal (1|dog). Models were fitted by maximum likelihood. Predicted population-level temporal trajectories (fixed effects only) were obtained over a continuous time grid spanning T0 to T8h (0 to 480 minutes, 5-minute intervals); T24h was excluded from the prediction grid due to the disproportionate time interval relative to the preceding measurements, which risks artefactual behaviour of the spline in this interval. The ten strain parameters included in this sensitivity analysis were: global radial strain (rStglobal: mean of the peak systolic radial strain of the 6 left ventricular segments), endocardial circumferential strain (cStendo: peak systolic circumferential strain assessed at the endocardial level), mid-myocardial circumferential strain (cStmid: peak systolic circumferential strain assessed at the mid-myocardial level), epicardial circumferential strain (cStepi: peak systolic circumferential strain assessed at the epicardial level), global circumferential strain (cStglobal: mean of peak cStendo, cStmid and cStepi), mean global longitudinal strain (lStglobalmean: mean of the mean peak systolic longitudinal strain of the 6 left ventricular segments assessed from the left apical 4-chamber, 5-chamber and 2-chamber views), mean basal longitudinal strain (lStbasalmean: mean of the peak systolic longitudinal strain averaged from basal septal and basal lateral segments across the left apical 2-, 4-, and 5-chamber views), mean mid-wall longitudinal strain (lStmidmean: mean of the peak systolic longitudinal strain averaged from mid septal and mid lateral segments across the left apical 2-, 4-, and 5-chamber views), mean apical longitudinal strain (lStapicalmean: mean of the peak systolic longitudinal strain averaged from apical septal and apical lateral segments across the left apical 2-, 4-, and 5-chamber views), and the base-to-apex longitudinal strain gradient, defined as the difference between lStbasalmean and lStapicalmean.

The pharmacodynamic Tmax was defined as the time point at which the spline-predicted population-level response reached its maximum absolute value within the T0–T8h observation window. It was estimated by identifying the peak of the predicted trajectory over a fine time grid with a one-minute resolution. Given the mathematical complexity of deriving analytical confidence intervals around a Tmax estimated from a spline model, bootstrap confidence intervals were computed using a cluster resampling approach to account for the repeated-measures structure of the data. At each of the 500 bootstrap iterations, five dogs were resampled with replacement from the original five animals, each contributing all their time points, and the spline model was refitted on the resampled dataset. The Tmax was extracted from each bootstrap replicate, and the 95% confidence interval was defined as the 2.5th and 97.5th percentiles of the resulting bootstrap distribution. A random seed was set prior to bootstrapping to ensure reproducibility of the results.

**Results**

The spline models identified a significant global effect of time for nine of the ten strain parameters examined (Table 1). Significant temporal trajectories were observed for rStglobal, cStendo, cStmid, cStglobal, lStglobalmean, lStmidmean, lStapicalmean, and the base-to-apex longitudinal strain gradient. The global effect of time failed to reach significance for cStepi (p=0.081) and lStbasalmean (p=0.44). These findings were fully concordant with the results of the primary categorical models reported in the main manuscript, in which cStepi showed only a marginal significant effect at a single time point (T4h), and lStbasalmean showed no significant change from baseline at any time point. The consistency between the two modelling approaches supports the robustness of the primary findings and confirms that the choice of treating time as a categorical variable did not materially affect the conclusions of the study.

**Table 1.** Global effect of time on strain parameters: likelihood ratio test comparing the natural cubic spline model to a time-invariant null model.

| **Variable** | **Chi2** | **P** |
| --- | --- | --- |
| rStglobal | 31.95 | <0.001 |
| cStendo | 30.64 | <0.001 |
| cStmid | 19.96 | <0.001 |
| cStepi | 6.74 | 0.081 |
| cStglobal | 21.31 | <0.001 |
| lStglobalmean | 27.86 | <0.001 |
| lStbasalmean | 2.70 | 0.44 |
| lStmidmean | 19.31 | <0.001 |
| lStapicalmean | 20.35 | <0.001 |
| Base-to-apex gradient | 11.20 | 0.011 |

*Chi², likelihood ratio test statistic; p, p-value for the global effect of time. A significant p-value indicates that the temporal trajectory of the parameter differs significantly from a time-invariant model. Degrees of freedom for all tests = 3.*

The estimated pharmacodynamic Tmax, defined as the time point at which the spline-predicted population-level response reached its peak, ranged from 166 to 195 minutes across all strain parameters, corresponding to the T2h–T4h interval identified in the primary categorical analysis (Table 2). For parameters with a significant and robust temporal effect, the bootstrap 95% confidence intervals around the Tmax were relatively narrow and consistent: rStglobal 177 min [171; 181], cStendo 166 min [157; 177], cStmid 170 min [157; 190], cStglobal 169 min [157; 186], lStglobalmean 182 min [170; 205], lStapicalmean 172 min [162; 187], and the base-to-apex gradient 168 min [141; 195]. For cStepi and lStmidmean, the confidence intervals were substantially wider ([153; 343] and [172; 245], respectively), reflecting greater inter-individual variability in the temporal trajectory of these parameters and the limited statistical power associated with the small sample size. For lStbasalmean, for which no significant global temporal effect was detected, the Tmax estimate was considered non-estimable, as the bootstrap confidence interval spanned the entire observation period [0; 201], indicating that the spline model failed to identify a consistent peak response for this parameter.

Taken together, these results demonstrate that the continuous spline modelling approach yields pharmacodynamic Tmax estimates that are fully consistent with the time points of maximal effect identified in the primary categorical analysis, while additionally quantifying the temporal trajectory of the pharmacodynamic response and confirming the absence of a meaningful temporal effect for lStbasalmean and, to a lesser extent, cStepi.

**Table 2.** Estimated pharmacodynamic Tmax of strain parameters derived from natural cubic spline models following intravenous pimobendan administration in five healthy awake Beagle dogs.

| **Variable** | **Tmax (min)** | **Lower 95% CI** | **Upper 95% CI** |
| --- | --- | --- | --- |
| rStglobal | 177 | 171 | 181 |
| cStendo | 166 | 157 | 177 |
| cStmid | 170 | 157 | 190 |
| cStepi | 174 | 153 | 343 |
| cStglobal | 169 | 157 | 186 |
| lStglobalmean | 182 | 170 | 205 |
| lStbasalmean | 182 | 0 | 201 |
| lStmidmean | 195 | 172 | 245 |
| lStapicalmean | 172 | 162 | 187 |
| Base-to-apex gradient | 168 | 141 | 195 |

*Tmax, time to peak pharmacodynamic effect estimated from the population-level temporal trajectory predicted by the natural cubic spline mixed-effects model. 95% CI, bootstrap 95% confidence interval around the Tmax estimate, derived from 500 cluster bootstrap resampling iterations. All strain values are expressed as absolute values.* 

The predicted population-level temporal trajectories estimated by the spline models are illustrated for all ten strain parameters in Figure 1.

**Figure 1.** Population-level temporal trajectories of strain parameters estimated by natural cubic spline mixed-effects models following intravenous pimobendan administration in five healthy awake Beagle dogs.

*Pimobendan (0.15 mg/kg IV) was administered immediately after the baseline (T0) measurements. Each colored line and its corresponding points represent an individual dog. The solid black line represents the population-level temporal trajectory predicted by the natural cubic spline mixed-effects model (fixed effects only). Vertical dotted lines indicate the seven measurement time points (T0, T30min, T2h, T4h, T6h, T8h). T30min is not labelled on the x-axis for clarity but is indicated by the first dotted vertical line. All strain values are expressed as absolute values. cStendo, peak systolic circumferential strain assessed at the endocardial level; cStepi, peak systolic circumferential strain assessed at the epicardial level; cStglobal, mean of peak cStendo, cStmid and cStepi; cStmid, peak systolic circumferential strain assessed at the mid-myocardial level; lStapicalmean, mean of the peak systolic longitudinal strain averaged from apical septal and apical lateral segments across the left apical 2-, 4-, and 5-chamber views; lStbasalmean, mean of the peak systolic longitudinal strain averaged from basal septal and basal lateral segments across the left apical 2-, 4-, and 5-chamber views; lStglobalmean, mean of the mean peak systolic longitudinal strain of the 6 left ventricular segments assessed from the left apical 4-chamber, 5-chamber and 2-chamber views; lStmidmean, mean of the peak systolic longitudinal strain averaged from mid septal and mid lateral segments across the left apical 2-, 4-, and 5-chamber views; rStglobal, mean of the peak systolic radial strain of the 6 left ventricular segments.*

**Conclusions**

The results of the natural cubic spline mixed-effects models were fully concordant with those of the primary categorical analysis. The global effect of time was significant for nine of the ten strain parameters examined, with the two non-significant parameters (lStbasalmean and cStepi) being the same as those showing absent or marginal effects in the primary analysis. This concordance confirms that treating time as a categorical variable in the primary models did not introduce bias or materially affect the conclusions of the study. The spline models additionally allowed estimation of a pharmacodynamic Tmax for each strain parameter. The estimated Tmax values were strikingly consistent across parameters, ranging from 166 to 195 minutes after injection, corresponding to the T2h–T4h interval.
